# Applying Cryo-X-ray Photoelectron Spectroscopy to Study the Surface Chemical Composition of Fungi and Viruses

**DOI:** 10.3389/fchem.2021.666853

**Published:** 2021-05-28

**Authors:** Andrey Shchukarev, Emelie Backman, Samuel Watts, Stefan Salentinig, Constantin F. Urban, Madeleine Ramstedt

**Affiliations:** ^1^ Department of Chemistry, Umeå University, Umeå, Sweden; ^2^ Department of Clinical Microbiology, Umeå University, Umeå, Sweden; ^3^ Umeå Centre for Microbial Research (UCMR), Umeå University, Umeå, Sweden; ^4^ Biointerfaces Lab, Empa, Swiss Federal Laboratories for Material Science and Technology, St. Gallen, Switzerland; ^5^ Department of Chemistry, Fribourg University, Fribourg, Switzerland

**Keywords:** cryo-XPS, virus, fungi, reference data, bacteriophage, surface chemistry, cell wall

## Abstract

Interaction between microorganisms and their surroundings are generally mediated *via* the cell wall or cell envelope. An understanding of the overall chemical composition of these surface layers may give clues on how these interactions occur and suggest mechanisms to manipulate them. This knowledge is key, for instance, in research aiming to reduce colonization of medical devices and device-related infections from different types of microorganisms. In this context, X-ray photoelectron spectroscopy (XPS) is a powerful technique as its analysis depth below 10 nm enables studies of the outermost surface structures of microorganism. Of specific interest for the study of biological systems is cryogenic XPS (cryo-XPS). This technique allows studies of intact fast-frozen hydrated samples without the need for pre-treatment procedures that may cause the cell structure to collapse or change due to the loss of water. Previously, cryo-XPS has been applied to study bacterial and algal surfaces with respect to their composition of lipids, polysaccharides and peptide (protein and/or peptidoglycan). This contribution focuses onto two other groups of microorganisms with widely different architecture and modes of life, namely fungi and viruses. It evaluates to what extent existing models for data treatment of XPS spectra can be applied to understand the chemical composition of their very different surface layers. XPS data from model organisms as well as reference substances representing specific building blocks of their surface were collected and are presented. These results aims to guide future analysis of the surface chemical composition of biological systems.

## Introduction

Since billions of years our planet is colonized by a huge amount of microorganisms that inhabit virtually all ecosystems and have forged livelihood of our world in decisive manner (Cavicchioli et al., 2019). A key factor determining the interactions between microorganisms and their habitat is the composition of the microbial surface. The term “microorganisms” generally includes viruses, bacteria, algae, and fungi. Thus, this group contains both prokaryotic and eukaryotic organisms, as well as virus particles from the realm viria. All of these exhibit differences in their surface chemistry relating to the structure and composition of their cell envelope, cell wall or particle surface. They also exhibit a large variation in size starting from viruses that are in the range of 20–500 nm (Encyclopedia Britannica, 2000) to fungal cells whose filaments can stretch dimensions of hundreds of micrometers (Cottier and Hall, 2020).

In order to characterize the chemistry of the outermost surface layers of microorganisms X-ray photoelectron spectroscopy (XPS) is an attractive technique due to its surface sensitivity, probing less than 10 nm into the material (Genet et al., 2008). This surface sensitivity enables analysis of the microbial surface without interference from the bulk of the microbial particle. XPS has been used for the analysis of microorganisms for several decades, with main focus on fungi and bacteria (van der Mei et al., 2000). Most of this work has been performed on freeze-dried specimens in order to give compatibility with the high vacuum of the spectrometer. Recently, we have been applying a cryogenic approach to XPS (cryo-XPS) where a biological sample is fast-frozen to liquid nitrogen temperatures prior to analysis and maintained at this temperature throughout the analysis time (Ramstedt and Shchukarev, 2016; Shchukarev and Ramstedt, 2017). Data from cryo-XPS has been shown to be in close agreement with data obtained from near-ambient pressure XPS for a reference bacterial strain (Kjærvik et al., 2021). Furthermore, we developed a model to predict the composition of lipid, polysaccharide and peptide (protein and/or peptidoglycan) at the surface of the cell from the C 1s spectrum (“the Umeå method”). Spectral components derived from multivariate analysis of a spectral library of Gram-negative bacteria and standards, representing building blocks for the bacterial cell wall, were used for these studies (Ramstedt et al., 2011). The model was also compared to existing data treatment methods relying on equation systems (Rouxhet and Genet, 2011; Ramstedt et al., 2014; Ramstedt et al., 2019; Hagberg et al., 2020). The two data treatment methods of the C 1s spectra showed similar results when applied to different datasets from bacterial cells, but with a systematic difference that appeared to be related to the ratio of lipid to peptide (Hagberg et al., 2020). The surface chemistries of bacterial cells, both Gram-negative (Ramstedt et al., 2011) and Gram-positive (Ramstedt et al., 2014; Ramstedt et al., 2019), as well as microalgae (Shchukarev et al., 2020) have been evaluated with this approach.

Here, we aim to investigate how well the methodologies developed for bacterial cells can be applied to understand the surface chemistry of fungal cells as well as bacterial viruses (bacteriophages). Using the spectral components to predict chemical composition of microalgae (Shchukarev et al., 2020), we recently discovered that the method does not fully account for polysaccharides with nitrogen containing functional groups or carboxylic acids, such as glucosamine and glucuronic acid. These substances represent building blocks for the eukaryotic cell wall in many microalgae and fungi. Hence, one of the aims of this study is to investigate to what extent our spectral components can be applied to characterize a broader range of microorganisms such as fungal cells and viruses that exhibit differences in surface structures compared to bacterial and algal cells. In order to test the methodology, we have chosen to work with two very different types of microorganisms, expecting that they would challenge the methodology in different ways. As model species of fungi, we selected *Candida albicans* and *Cryptococcus neoformans,* for the study of their cell wall composition. The former was chosen to enable comparisons of fungal cells that are present as spherical yeasts or filamentous hyphae. The latter for comparisons of yeasts with and without capsule (Lopes et al., 2019). Furthermore, we selected two viruses in the form of bacteriophages, a non-enveloped, MS2, and an enveloped, Phi6, for studies of viral surface chemistry (Pitol et al., 2017; Watts et al., 2020).

This leads into the further goals of this study, namely i) the proof-of concept analyses of the aforementioned microorganisms, ii) continued evaluation of the model using spectral components, and iii) providing ample reference data for the substances that represent building blocks for the microbial surface, as well as for the intact microorganisms themselves.

### Fungal Surface Chemistry

**FIGURE 1:**
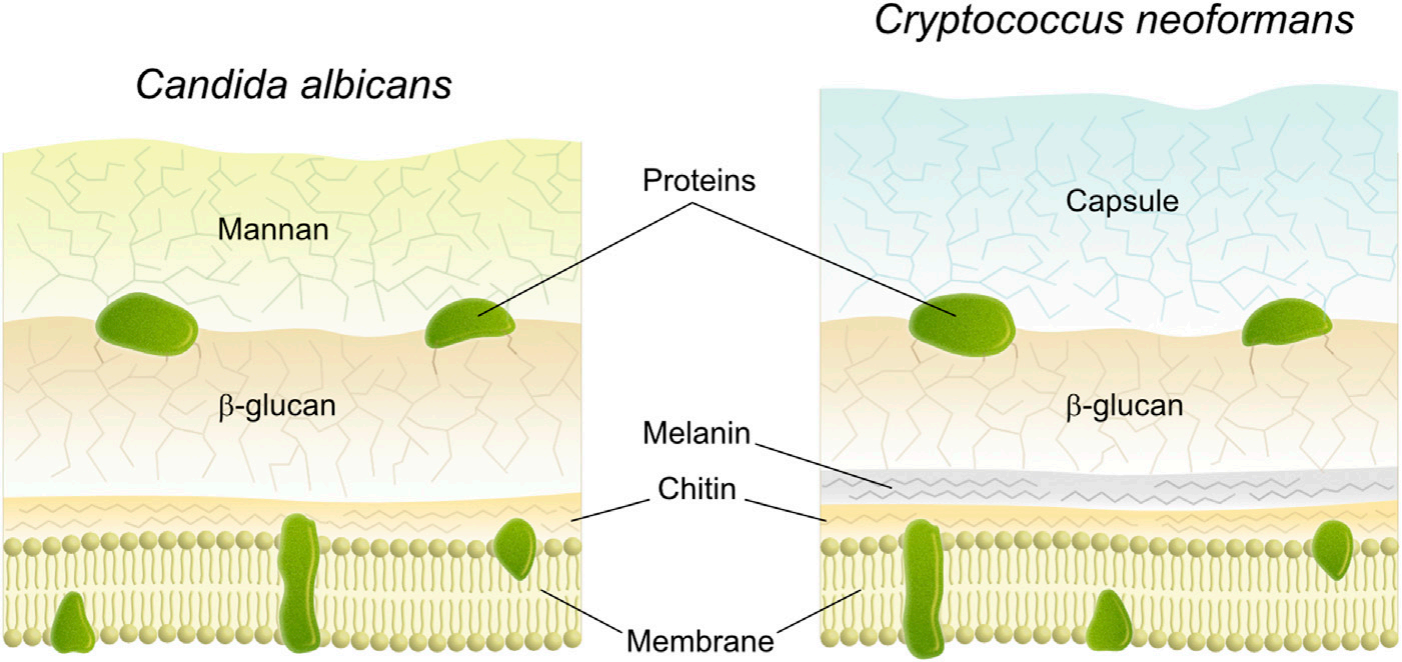
Schematic drawing of the cross section of the cell wall of fungi a) *Candida albicans* and b) *Cryptococcus neoformans* (Gow et al., 2017).

The chemistry of the fungal cell wall is very dynamic and responsive to environmental cues, but its overall composition can to some degree be related to their genus in the taxonomy (Peberdy, 1990; Gow et al., 2017). In general, the largest building block in the fungal cell wall consist of different types of polysaccharides. Water-insoluble chitin (polymer of N-acetyl-D-glucose) and beta-linked glucans (polymer of D- glucose) are important to maintain the structural shape of the cell (Figure 1) (Peberdy, 1990; Gow et al., 2017). In addition, there are amorphous water-soluble polysaccharides mainly consisting of polymers from glucose (Peberdy, 1990). Approximately 50–60% dry weight of the cell wall consist of beta-glucan (Lima et al., 2019) and chitin is generally present at lower abundance (Bowman and Free, 2006). Apart from these, (mannosylated glyco)proteins, lower amounts of lipids, polymers from galactosamine and glucosamine, melanins as well as polyuronides may be present (Peberdy, 1990; Lima et al., 2019). The cell wall most likely has a layered structure in which the scaffold comprises the inner layers whereas the more amorphous outer layers embed this scaffold (Figure 1). The outer layer exhibit most of the species-dependent cell-wall chemistry (Gow et al., 2017; Lima et al., 2019) where for example *Saccharomyces, Candida* and *Pneumocystis* have outer layers containing mannosylated proteins (mannan), while *Aspergillus* have outer protective layers of hydrophobic proteins (hydrophobins) as well as melanin pigments (Gow et al., 2017). Fungi may also be shielded by a well-organized gelatinous capsule, as in *Cryptococcus.* This capsule is made from galatoxylomannan and glucoronoxylomannan, where the latter makes up the bulk of the capsule (90 mass%) and consist of mannan with glucuronic acid and xylose as well as branches of acetyl groups (Gow et al., 2017). The cell wall of fungi, furthermore, appears to be adaptive to the environment. Thus, *C. albicans* have been described to alter its cell wall composition and thickness following changes e.g., in temperature, pH, oxygen, and nutrient levels (Lopes et al., 2018; Cottier and Hall, 2020). For this study, *C. albicans* was chosen as it is dimorphic and can reversibly switch morphology from yeast to hyphal forms upon various environmental cues, such as pH, temperature and presence of serum (Kim and Sudbery, 2011). *C. albicans* uses hyphal growth to penetrate tissue and to disseminate during severe forms of candidiasis in immunocompromised individuals (Naglik et al., 2019). *C. neoformans* in contrast is a yeast not able to form filaments. However, it can protect itself from phagocytosis with the help of an amorphous capsule surrounding the cell wall (Zaragoza, 2019). To dissect surface chemistry with and without the capsule we here took advantage of wild type (WT) and related acapsular mutant strains.

Rouxhet et al. performed a substantial amount of work studying fungal and bacterial cells with XPS in the eighties and nineties using carefully freeze-dried organisms (van Haecht et al., 1982; Amory et al., 1988; Amory et al., 1988; Mozes et al., 1988; Mozes et al., 1989; James, 1991; Mozes et al., 1991; Rouxhet et al., 1994; Dengis and Rouxhet, 1996; Dengis et al., 1996; Rouxhet et al., 2008; Rouxhet and Genet, 2011). The work included systematic studies of artifacts that may arise due to poor temperature control during sample pre-treatment/drying and leading to increased amounts of lipid-like substances at the surface in fungal cells (Dengis and Rouxhet, 1996) similar to what we have observed for bacterial cells (Ramstedt et al., 2014). The focus of most of these fungal analyses were on cells from the species *Saccharomyces*, but a few studies of the yeast form of *C. albicans* have been reported (van der Mei et al., 2000). XPS has also been used to study uptake of metals into lyophilized micro and macro-fungi (Ding et al., 2015; Song et al., 2016; Song et al., 2016; Zhao et al., 2016) or interactions between fungal hyphae from *Aspergillus aculeatus* and graphene oxide (Zhang et al., 2016), whereas Dague et al. (2008) combined XPS with AFM and ToF-SIMS to study the surface of freeze-dried *Aspergillus fumigatus*.

### Virus Surface Chemistry

**FIGURE 2:**
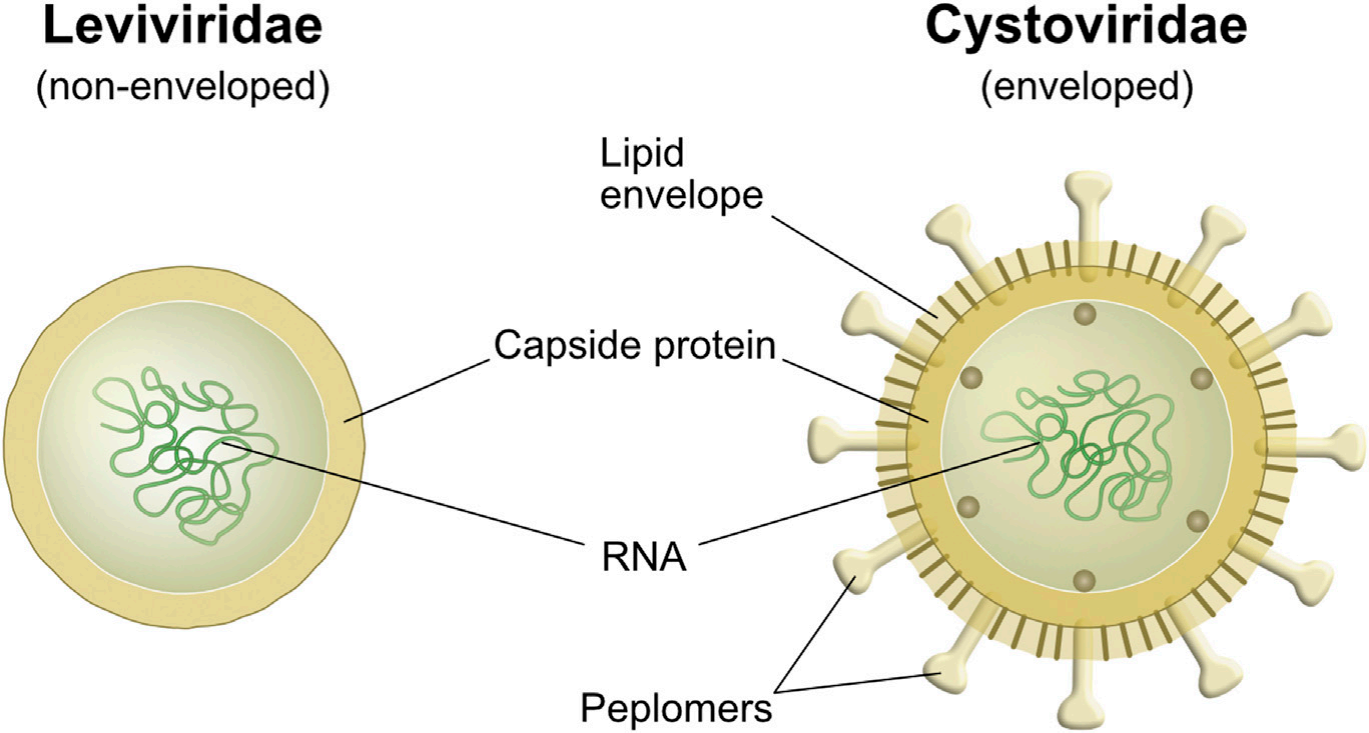
Schematic drawing of the cross section of viral particles from a) Leviviridae and b) Cystoviridae (King et al., 2011). Leviviridae represents a non-enveloped phage and Cystoviridae an enveloped phage with spikes/peplomers protruding through the lipid envelope.

The two main building blocks of viruses are proteins and nucleic acids. Beside these, a lipid membrane encapsulates enveloped viruses (Figure 2). Large variations in virus structure and dimensions are found between taxonomic groups. The viral proteins are encoded in the RNA or DNA carried by the viral particles (Fenner et al., 1987). These proteins form the glycosylated surface structures called spikes (peplomers) or consist of the non-glycosylated matrix proteins that give stability to the viral particle, forming the protein cage (Fenner et al., 1987). Viruses with a lipid envelope display differences in the lipid composition that are linked to the type of host cell that they infect to propagate (Lucas and Knipe, 2002; Buchmann and Holmes, 2015). Mammalian viruses typically exhibit a lipid envelope originating from internal membranes in the cell (e.g., nuclear envelope, endoplasmic reticulum, Golgi apparatus) or from the plasma membrane (Fenner et al., 1987). The lipid content for these types of enveloped viruses may be up to a third of the dry weight and generally consist of roughly equal parts of phospholipids and cholesterol (Fenner et al., 1987). The envelope probably has evolved as an adaptation to the host allowing for membrane fusion, whereas other infection routes are used by non-enveloped viruses (Buchmann and Holmes, 2015). Viruses that infect bacteria are called phages and exist both as enveloped and non-enveloped structures. Phages with envelope, for example the *Pseudomonas* phage Phi6, obtain their lipids from the plasma membrane of the bacterium (King et al., 2011). The Phi6 phage has a spherical shape, a diameter of around 85 nm, and comes from the family of the *Cystoviruses* (King et al., 2011). In the literature, the Phi6 has been used as a surrogate for lipid-enveloped mammalian viruses (Pitol et al., 2017). Phages without envelope consist of proteins and nucleic acids (King et al., 2011). Examples of phages without envelope are the commonly used lab phage, lambda phage, from the family *Siphoviridae*. It has an icosahedral “head” with a diameter of around 60 nm and thin tubular tail with a length of around 150 nm. Other non-enveloped phages are the MS2 and Qbeta phages, from the family *Leviviridae,* which consist of phages around 26 nm in diameter that have an icosahedral structure and propagate in *Escherichia coli* (Kuzmanovic et al., 2003; King et al., 2011; Watts et al., 2020).

When it comes to analysis of viruses and phages using XPS, very few studies exist and they mainly deal with applications in bioengineering using phages as a tool (Tawil et al., 2013; Tawil et al., 2015; Rho et al., 2018). To our knowledge, XPS has not previously been used in studies focusing on understanding the surface chemistry of viruses.

## Material and Methods

Commercially available solid (powder) reference samples of: cholesterol (Sigma), melanine from *Sepia officinalis* (Sigma), mannan from *Saccharomycs cerevisiae* (Sigma), DNA from salmon testes (Sigma), RNA from torula yeast (Sigma), galactoseamine (Merck), N-acetyl galactoseamine (Merck), soy phospholipids (Merck), as well as building blocks of DNA and RNA, i.e., uracil (Merck), thymine (Koch), adenine (CHR), guanine (Sigma), and cytosine (Sigma) were all analyzed at room temperature, without further purification, and after gentle homogenization in a mortar.

Lysogeny Broth (LB) was prepared by adding 10 g/L of Tryptone (Fluka Analytics), 1 g/L of select yeast extract (Sigma life science), 8 g/L of NaCl (≥99.5% purity, Sigma-Aldrich Chemie GmbH), 0.3 g/L of CaCl_2_ (97% purity, Fluka Analytics), 1 g/L Dextrose (Biotechnology grade, Amresco) and 2 mg/L Streptomycin (Fluka Chemie GmbH) in ultra-pure water.

The virus dilution buffer (i.e phosphate buffered saline, PBS) was prepared by adding 0.78 g/L NaH_2_PO_4_ 2H_2_O (≥98.0% purity, Sigma-Aldrich Chemie GmbH, Steinheim, Germany) and 0.58 g NaCl (≥99.5% purity, Sigma-Aldrich Chemie GmbH, Steinheim, Germany) in ultra-pure water. The pH was equilibrated to seven using NaOH (≥99% purity, Carl Roth GmbH, Karlsruhe, Germany) and HCl (ACS reagent grade, Sigma-Aldrich, Buchs, Switzerland).

### Fungal Growth

*Candida albicans* SC5314 (wild type) (Gillum et al., 1984), *Cryptococcus neoformans* B3501 (wild type) and *Cryptococcus neoformans* cap59 (capsule mutant of B3501) (Zaragoza et al., 2008) were incubated in synthetic complete dropout medium without uracil (SC medium) for 16 h at 30°C (Lopes et al., 2019). To induce hyphal growth, *C. albicans* was incubated for 16 h at 37°C. Cells were pelleted by centrifugation at 3,000 × g for 5 min. Cell pellets were subsequently washed two times with PBS followed by one time with milliQ water. For pelleting after washes, tubes were centrifuged at 5,000 × g for 3 min. The entire liquid above cell pellets was carefully removed before the samples was placed on ice until measurement.

### Virus Propagation and Purification

MS2 (DSMZ 1,367, DSMZ, Germany) was replicated in *Escherichia coli* strain W1485 (DSMZ 5,695, DSMZ, Germany). *E. coli* was incubated in LB broth, described above, at T = 37°C at 80 rpm (Multitron, Infors-ht, Germany). When the bacteria culture reached an optical density at 600 nm of 0.04 (Genesys 6, ThermoSpectronic, United States), it was infected with MS2 at a ratio to *E. coli* of 0.1 (multiplicity of infection). The infected bacteria were further incubated for 4–5 h at T = 37°C without shaking (Multitron, Infors-ht, Germany).

Phi6 (DSMZ 21,518, DSMZ, Germany) was replicated in *Pseudomonas syringae* (DSMZ 21,482, DSMZ, Germany). *P. syringae* was incubated in 2 L of Tryptic Soy Broth (TSB) broth (Sigma 220,952, Sigma-Aldrich Chemie GmbH, Steinheim, Germany) at T = 25°C, 80 rpm (Multitron, Infors-ht, Germany). When the bacteria culture reached an optical density of 0.1 (Genesys 6, ThermoSpectronic, United States), it was infected with 500 µl of Phi6 at 10^9^ PFU/ml. The infected bacteria were further incubated overnight at T = 25°C, 80 rpm (Multitron, Infors-ht, Germany).

For both bacteriophages, the bacterial debris were removed by centrifugation at 2,600 × g for 20 min (Eppendorf 5810R, Eppendorf Germany). The phage containing supernatant was filtered with 0.22 µm sterile filters (MillexGP, Millipore, Ireland) to remove any remaining bacterial debris. For MS2, the flow through was then concentrated from 7 L to approximately 5 ml with centrifugal filters (100 kDa Amicon Ultra-15 centrifugal filters, Millipore, Ireland). The solution was washed with at least 5 L of PBS, composition described above, at pH = 7.0 by multiple 13–15 ml washing steps in the centrifugal filters. The solution was finally filtered through 0.1 µm sterile filters (MillexVV, Millipore, Ireland), in agreement with the virus purification method previously reported (Armanious et al., 2016). The final volume was adjusted to achieve a final MS2 concentration of 10^14^ PFU/ml corresponding to about 1 mg/ml.

For Phi6, the flow through was concentrated from 2 L to 1 ml and washed with 1 L PBS using centrifugal filters (100 kDa Amicon Ultra-15 centrifugal filters, Millipore, Ireland). The suspension was further purified by glucose gradient (5–20% w/v) ultracentrifugation at 47,808 × g for 50 min at 15°C (Sorvall RC 6 Plus Superspeed Centrifuge, Thermo Scientific, Waltham, MA, United States). The 5 ml 15% glucose fraction, containing the Phi6, was recovered. The Phi6 particles were then concentrated to 200 µl and washed with 5 L PBS using centrifugal filters (100 kDa Amicon Ultra-15 centrifugal filters, Millipore, Ireland) in order to eliminate the glucose. The volume was adjusted to achieve about 10^12^ PFU/ml that correspond to about 1 mg/ml. This method is a combination of two previously reported methods for Phi6 purification (Pitol et al., 2017; Bamford et al., 1995).

### X-ray Photoelectron Spectroscopy Analyses

XPS analyses were performed on a Kratos Axis Ultra DLD spectrometer with a monochromated Al K*α* source operated at 150 W. Survey spectra were acquired with an analyzer pass energy of 160 eV and high-resolution spectra at 20 eV. A hybrid lens system with a magnetic lens, providing an analysis area of 0.3 mm by 0.7 mm, and the built-in charge neutralizer system were used for all measurements. The majority of samples containing microorganisms were analyzed “floating” (without ground) to avoid differential charging initially observed for some of the fungal samples (two of the filamentous *C. albicans* hyphae samples and one *C. albicans* yeast sample). Data from sample replicas with signs of differential charging were discarded and not used further. Standard references were analyzed at room temperature while cells were analyzed at liquid nitrogen temperature (−160°C). During analyses, under liquid nitrogen cooling, the pressure inside the analysis chamber was generally between 1E-8 and 1E-9 Torr.

Fungal samples were analyzed using the same approach as previously has been described for bacterial cells (Ramstedt et al., 2011; Ramstedt and Shchukarev, 2016). Freshly grown cultures of fungal cells, washed on the morning of analysis, were kept on ice. A small portion (∼20 μl) of the wet cell pellet was transferred with an automatic pipette to the sample holder at room temperature, the pellet on sample holder was fast-frozen in the sample loading chamber of the spectrometer and analyzed under liquid nitrogen cooling (−160°C) in the sample analysis chamber.

Phages were analyzed in solution according to procedures described for outer-membrane vesicles and solution compounds (Hagberg et al., 2020). Bacteriophages, Phi6 and MS2, were stored suspended in PBS at +4°C, and analyzed as suspensions. A volume of 15–20 µl suspension containing approximately 10^10^ plaque forming units (PFU) for Phi6, or 10^12^ PFU for MS2, was transferred with an automatic pipette and fast-frozen directly onto a pre-cooled sample holder (−160 to −170°C) inside the loading chamber of the XPS. Thereafter, the frozen sample drop was transferred into the analysis chamber and analyzed under liquid nitrogen cooling (−160°C).

### Data Processing

Processing of the spectra was accomplished with Casa XPS (Gaussian-Lorentzian peak shapes GL30, and Shirley background) and Vision2 Kratos software. The binding energy (BE) scale of all spectra was referenced to the C 1s line of aliphatic carbon, set at 285.0 eV, unless specifically stated otherwise. The fitting was performed using as few components as possible and without restricting the peak parameters, unless otherwise specified.

The contribution of protein, lipid and polysaccharide in C 1s XPS spectra was evaluated using a previously described method, “the Umeå method,” predicting the percentage of C atoms from each of the three substances in relation to total C atoms at the surface (Ramstedt et al., 2011; Ramstedt and Shchukarev, 2016). This method uses previously defined spectral envelopes obtained from multivariate curve resolution of a collection of bacterial and reference spectra assuming that the collection of spectra could be interpreted as a linear combination of three spectral components. The spectral shapes obtained during that analysis suggested that they closely describe the content of lipid-like substances, substances with peptide bonds (protein and peptidoglycan) and polysaccharides (Ramstedt et al., 2011). These spectral components were used without adjustment of peak width or shape along the binding energy scale, i.e., the only parameter fitted was the intensity of the components giving differences in relative area covered for respective C 1s spectrum. The overall fit represents a linear combination of the three spectral components.

## Results and Discussion

XPS analyses were performed both on intact microorganisms as well as their individual building blocks including RNA, DNA, phospholipids, and polysaccharides. This approach was chosen to construct a reference library that may later aid in the interpretations of data from the intact microorganisms. Furthermore, the signatures of these building blocks were expected to aid in estimates of information depth for the microbial samples using cryo-XPS.

### Building Blocks for Fungal Cell Wall or Virus Particles

In order to investigate the individual substances that build the wall in fungi and the envelope in viruses, we analyzed a number of references and compared their elemental composition with the theoretical composition based on the chemical structure of the compounds (Tables 1, 2 and Supplementary Table S1).

**TABLE 1:** Theoretically and experimentally obtained atomic percentage (at %) of elements at the surface of reference samples.

Fungal references	Theory (at %)						Experiment (at %)					
	C	N	O	P	Na	Cl	C	N	O	P	Na	Cl
Melanin	75	8	17				64	12	19		2	3
Mannan	55		45				65	3	32		0	
Galactoseamine HCl	46	8	38			8	52	7	35			6
N-acetyl galactoseamine	53	7	40				57	6	36			

Virus references	Theory (at %)						Experiment (at %)					
	C	N	O	P	Na	Cl	C	N	O	P	Na	Cl
Cholesterol	96		4				97		3			
Phospholipids	81		17				80	1	17	1		
RNA^a^	45	17	33	5			50	14	32	4		
DNA^a^	48	18	29	5			52	15	25	4		

a Average of idealized structure consisting of equal amounts of nucleotides.

**TABLE 2:** Data from fitting of C1s spectra from reference samples using Gaussian-Lorentzian (GL30) peak shapes and spectral components.

Fungal reference	Theory (at %)			Exp (at %)			% of tot C		
	CH	CO/CN	CON/COO	CH	CO/CN	CON/COO	peptide	lipid	polysaccharide
Melanin	56	22	22	53	22	25	80	9	11
Mannan		83	17	28	55	16^b^	23	23	54
Galactoseamine HCl		83	17	8	73	19	15	0	85
N-acetyl galactoseamine	13	63	25	17	56	27	51	0	49

Virus reference	Theory (at %)			Exp (at %)			% of tot C		
	CH	CO/CN	CON/COO	CH	CO/CN	CON/COO	peptide	lipid	polysaccharide
Cholesterol	96	6		81	19		27	73	0
Phospholipids	87	8	5	64	32	4^a^	8	82	10
RNA				17	55	29^b^	38	0	62
DNA				35	43	22^b^	42	21	37

a = Intensity at 289.1 eV.

b = Summed intensity at 288.0–288.2 and 289.2–289.7.

**FIGURE 3:**
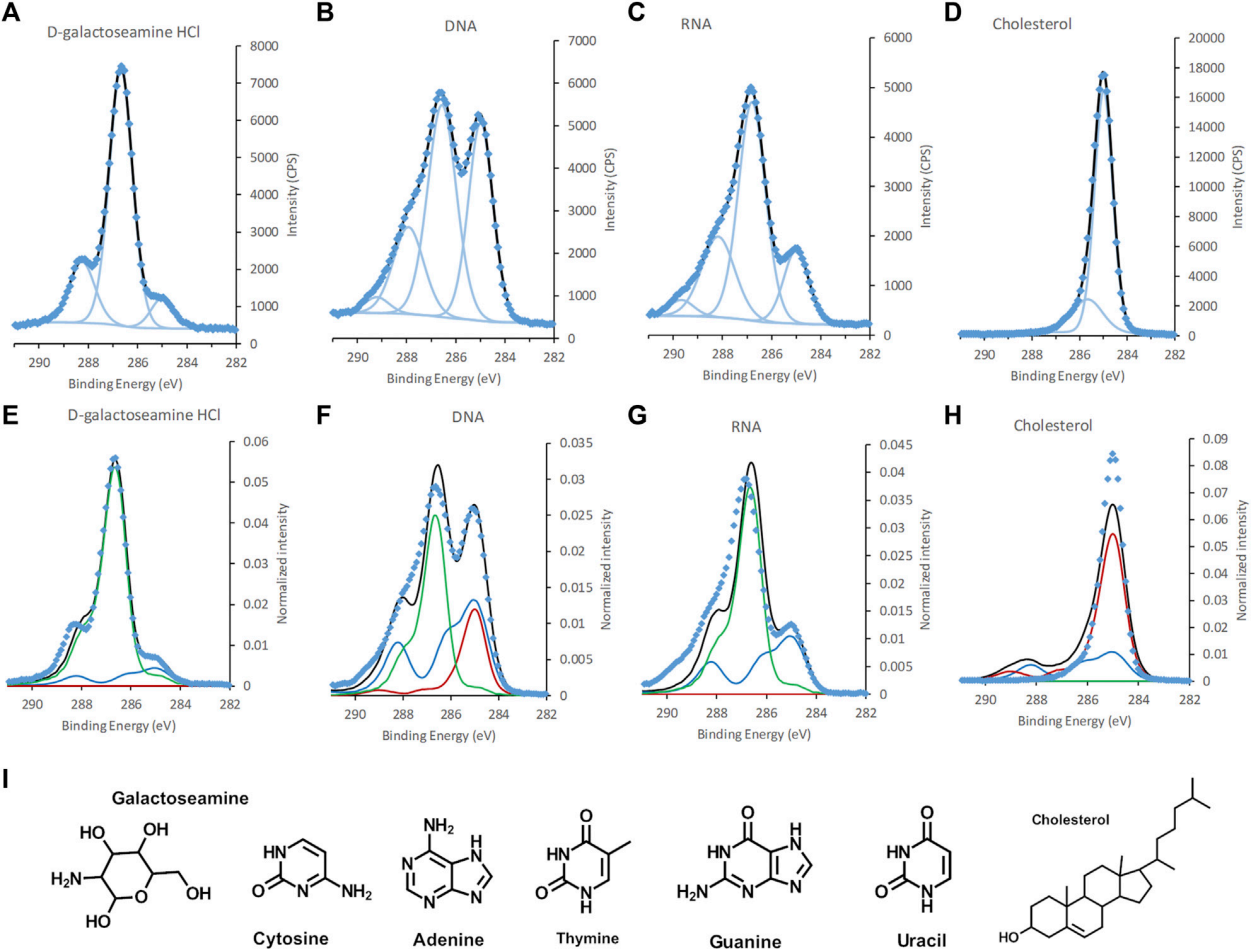
C 1s spectra of, **(A,E)** galactoseamine, **(B,F)** DNA, **(C,G)** RNA, and **(D,H)** cholesterol, **(A–D)** standard samples fitted with GL30 peakshapes and **(E–H)** with spectral components. **(I)** Chemical structure of galactoseamine, nucleobases in DNA and RNA, and cholesterol. Data is represented by blue diamonds, the fit by a solid black line, GL30 peak shapes are represented by light blue lines in figures **(A–D)**, and in **(E–F)** the blue line represents protein-like substance, green line polysaccharides and red line lipid-like substances.

For the building blocks of the fungal cell wall, we observed larger differences between the theoretical and experimental percentages for natural substances purified from organisms (mannan and melanin) than for the synthetic ones, which may indicate higher purity after synthesis than after extraction (Table 1). However, the experimental elemental compositions agree with theory within the estimated (<10%) error of XPS. The fit between the spectral components and the C 1s spectra of reference substances were overall good (Figure 3 and Supplementary Figure S1). However, some discrepancies were observed for a few building blocks (Figure 3). Previous studies of algal cell-wall building blocks showed that the model did not explain features around 287 and 289 eV, originating from amines in glucosamine and carboxylic groups in glucuronic acid building blocks. Thus, it is not surprising that the model did not fully explain a shoulder at 289.0 eV for galactose amine (Shchukarev et al., 2020). Both glucosamine and galactosamine are building blocks of the fungal cell wall. Thus, from the analysis of building blocks we can expect that the model may deviate in the same way for fungal cells as it does for algal cells (Shchukarev et al., 2020).

The elemental composition obtained for viral building blocks also agreed well with the theoretical percentage, within the expected measurement error (Table 1). For the DNA and RNA standards, data on the exact percentages of each nucleotide were not present, however, an estimate of equal amounts of the constituent bases agreed fairly well with the experimental data. When spectral components were fitted to the C 1s spectra of viral reference substances, a deviation between the model and the spectral intensity can be seen for DNA and RNA (Figure 3). Further analyses showed that this “unexplained intensity” originated from nucleobases with C atoms at different binding-energy positions from that of, i.e., carboxylic acids, ester and peptide groups existing in the idealized spectral components. For uracil, thymine, and cytosine the peak assigned to [N-(C = O)-N] was observed at 289.5, 289.4, and 288.7 eV, respectively, (Figure 3I, Supplementary Figure S2; Supplementary Table S1). These positions were slightly higher for two of the bases than the reported chemical shift of 3.84 eV (i.e. 288.84 eV) (Beamson and Briggs, 1992). For guanine the peak assigned to [N-(C=N)-N] was observed at 288.8 eV and could, thus, not be resolved from the C in the peptide bond at 288.2 eV. Thus, these data suggest that C atoms in nucleobases are the origin of the unexplained intensities observed in the DNA and RNA standards when fitted with spectral components (Supplementary Figure S2). For cholesterol, the spectral components over-fit the spectra and the model includes a peptide component (Figure 3; Table 2). The reason for this may be either asymmetry of the peaks, or reflects the higher intensity at 285.7 eV obtained experimentally compared to theoretical predictions (Table 2). From the analyses of the reference samples, it becomes clear that the percentages of spectral components should be interpreted with care (Table 2). Samples with large amounts of cholesterol at the surface would be predicted, by the model, as lipid with a small amount of peptide. Furthermore, the model would interpret the presence of DNA or RNA in a microbial sample as a mixture of peptide and polysaccharide in addition to unexplained intensity relating to the nucleobases. The reason for this erroneous prediction is that no spectral component is present that would capture the unique profiles of the nucleobases. Instead, the atoms with similar chemical environment as lipids, peptides and polysaccharides in the nucleic acids are interpreted as such substances. Thus, in order to correctly predict the content of RNA or DNA at the surface of a sample, new spectral components would need to be derived using the same approach that was previously used to obtain the current three spectral components (Ramstedt et al., 2011). Such development would be necessary for more accurate analyses of biological samples where nucleic acids are expected to be present in significant amounts at the surface of the sample. The need for such model with four spectral components would be indicated by the appearance of unexplained intensities when using only three spectral components, as shown above.

### Fungal Samples

From the analysis of fungal samples, we aimed to investigate differences in surface chemistry between cells in yeast and hyphal forms of *C. albicans*, as well as between a wild type (WT) *C. neoformans* yeast with capsule and a mutant lacking the capsule.

**FIGURE 4:**
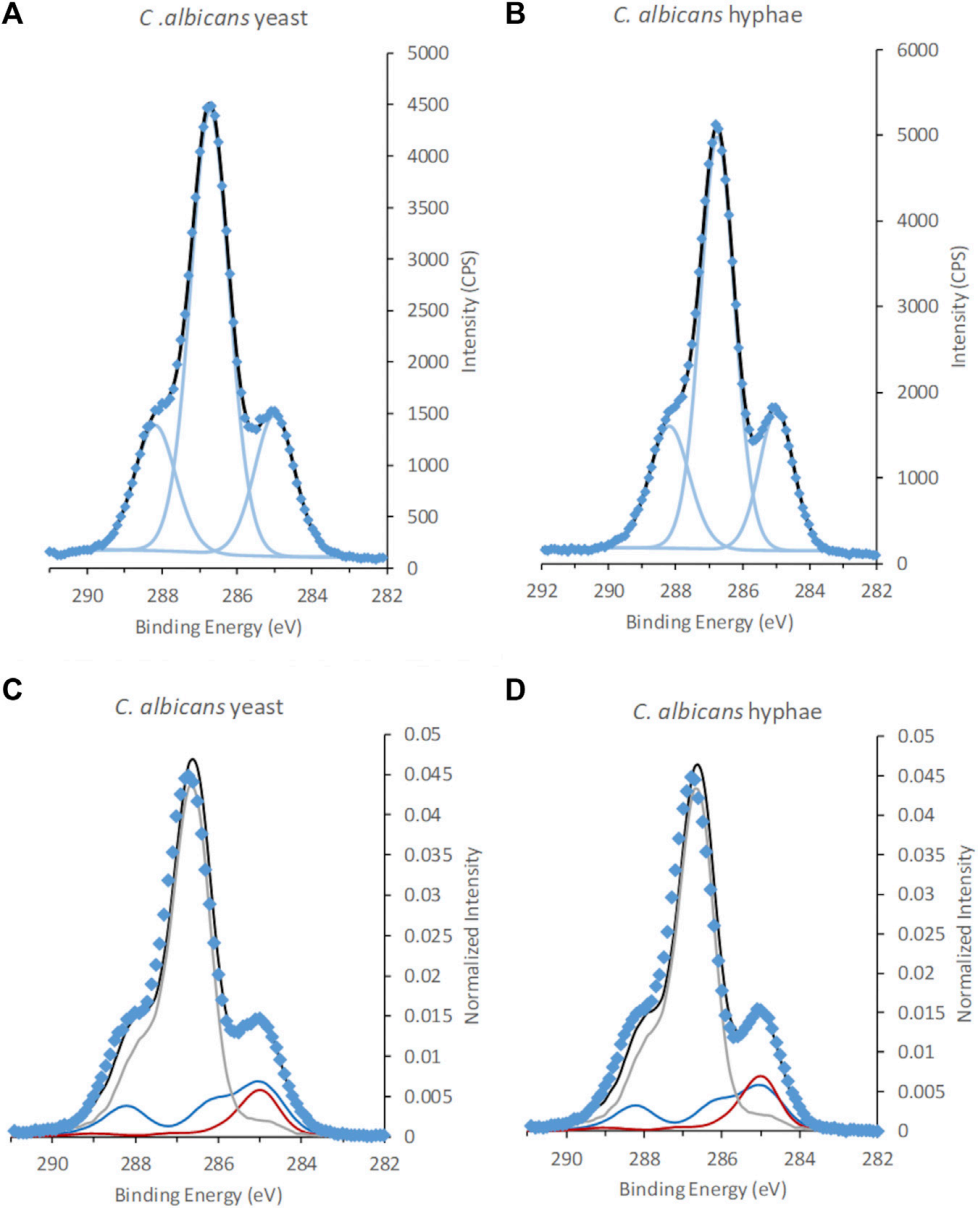
XPS C 1s spectra of *C. albicans* in the form of **(A,C)**, yeast and hyphae **(B,D)**. Spectra in **(A)** and **(B)** are fitted using traditional fitting of Gaussian-Lorentzian (GL30) peak shapes, **(C,D)** fitted using spectral components. Data is represented by blue diamonds, the fit by a solid black line, GL30 peak shapes are represented by light blue lines in figures **(A,B)**, and in **(C,D)** the blue line represents protein-like substance, grey line polysaccharides and red line lipid-like substances.

No significant differences in surface chemistry could be observed between the *C. albicans* cells in yeast and hyphal form (Figure 4; Table 3; Supplementary Figure S3; Supplementary Table S2). The C 1s spectra of *C. albicans* was dominated by a large peak at 286.7–286.8 eV assigned to C-O and C**-**N. In addition, two smaller intensities were observed at 285.0 and 288.2 eV, assigned to aliphatic carbon and carbon in peptide bonds, respectively. The N/C ratio was overlapping between the two morphological forms (yeast N/C = 0.05 ± 0.01 vs. hyphae N/C = 0.04 ± 0.01). Very low levels of Na^+^ and K^+^ counter ions were detected, whereas Cl^−^ was below the detection limit, indicating that excess salts were mostly removed during the last wash with water during sample preparation. The remaining fraction of counter ions indicates that the surface of the *C. albicans* cells had a slight negative charge. Literature data of freeze dried samples from *C. albicans* reports varying N/C ratios from 0.005–0.046, and P/C of 0.000–0.025 (van der Mei et al., 2000). Our dataset exhibits N/C ratios at the higher end of this range, and a P/C ratio (P/C = 0.007) within the range reported previously. Since anions such as Cl^−^ were absent at the negatively charged surface of *C. albicans* samples, i.e., removed by the water wash, this P was assigned to phosphonate groups at the surface.

**TABLE 3:** Average ratios of total N (N_tot_) and C1s GL30 peak shapes (BE position in square parenthesis) to total C (C_tot_), as well as predicted substance composition as % of total C atoms at the surface from fitting of C1s spectra (st dev in parenthesis).

	N_tot_	C [285.0]	C [286.5]	C [288.2]	C [289.5]	peptide	Lipid	polysaccharide
	/C_tot_	/C_tot_	/C_tot_	/C_tot_	/C_tot_			
Fungi								
*C. albicans* yeast	0.05	0.20	0.60	0.19		19	13	69
(*n* = 3)	(0.01)	(0.00)	(0.02)	(0.02)		(5)	(3)	(2)
*C. albicans* hyphae	0.04	0.19	0.60	0.21		20	11	69
(*n* = 3)	(0.01)	(0.04)	(0.02)	(0.02)		(9)	(10)	(2)
*C. neoformans* mut	0.11	0.32	0.48	0.19	0.01	49	10	42
(*n* = 3)	(0.03)	(0.05)	(0.07)	(0.03)	(0.01)	(11)	(2)	(11)
*C. neoformans* WT	0.08	0.21	0.50	0.24	0.06	51	0	49
(*n* = 5^a^)	(0.03)	(0.03)	(0.04)	(0.05)	(0.02)	(7)	(0)	(7)
Phages								
Phi6	0.14	0.42	0.39	0.18	0.01	48	24	28
(*n* = 3^b^)	(0.01)	(0.02)	(0.02)	(0.00)	(0.00)	(5)	(5)	(0)
MS2	0.24	0.43	0.31	0.24	0.02	81	6	14
(*n* = 3^b^)	(0.01)	(0.01)	(0.05)	(0.07)	(0.02)	(2)	(1)	(1)

a Five replicas from four different biological batches/days.

b Replica from the same large batch of phages.

The outermost layer of *C. albicans* cells has been described to consist of mannan and proteins (Gow et al., 2017). Tables 2, 3 show differences between the surface chemical composition of the mannan standard and the *C. albicans* cell wall relating both to an increased content of polysaccharide and a decrease in lipid for the cells. This may indicate presence of additional constituent within the information depth of cryo-XPS, or may relate to differences in mannan composition between *Saccharomyces* and *Candida*. The layer thickness of mannan at the surface of *C. albicans* has been reported to be around 50 nm (Lenardon et al., 2020). Thus, even though this thickness has been described to vary following environmental cues (Cottier and Hall, 2020), we conclude that XPS only probes the outermost part of the mannan layer at the surface of *C. albicans*. In contrast to C 1s spectra from microalgae, no unexplained shoulders originating from glucuronic acid or glucosamine were observed when fitting *C. albicans* spectra with the three spectral components (Figure 4), indicating that substances with these building blocks were present below the information depth of XPS (Shchukarev et al., 2020). The cell wall composition with respect to protein profile and content have been described to change between the two forms resulting in an increased protein content in the hyphal cell wall (Chaffin, 2008; Martínez-Gomariz et al., 2009). Such changes in proteome would not be detected using XPS except if they resulted in a significant change in the overall protein content, and occurred within the information depth of XPS, i.e., in the outermost nm of the surface.

**FIGURE 5:**
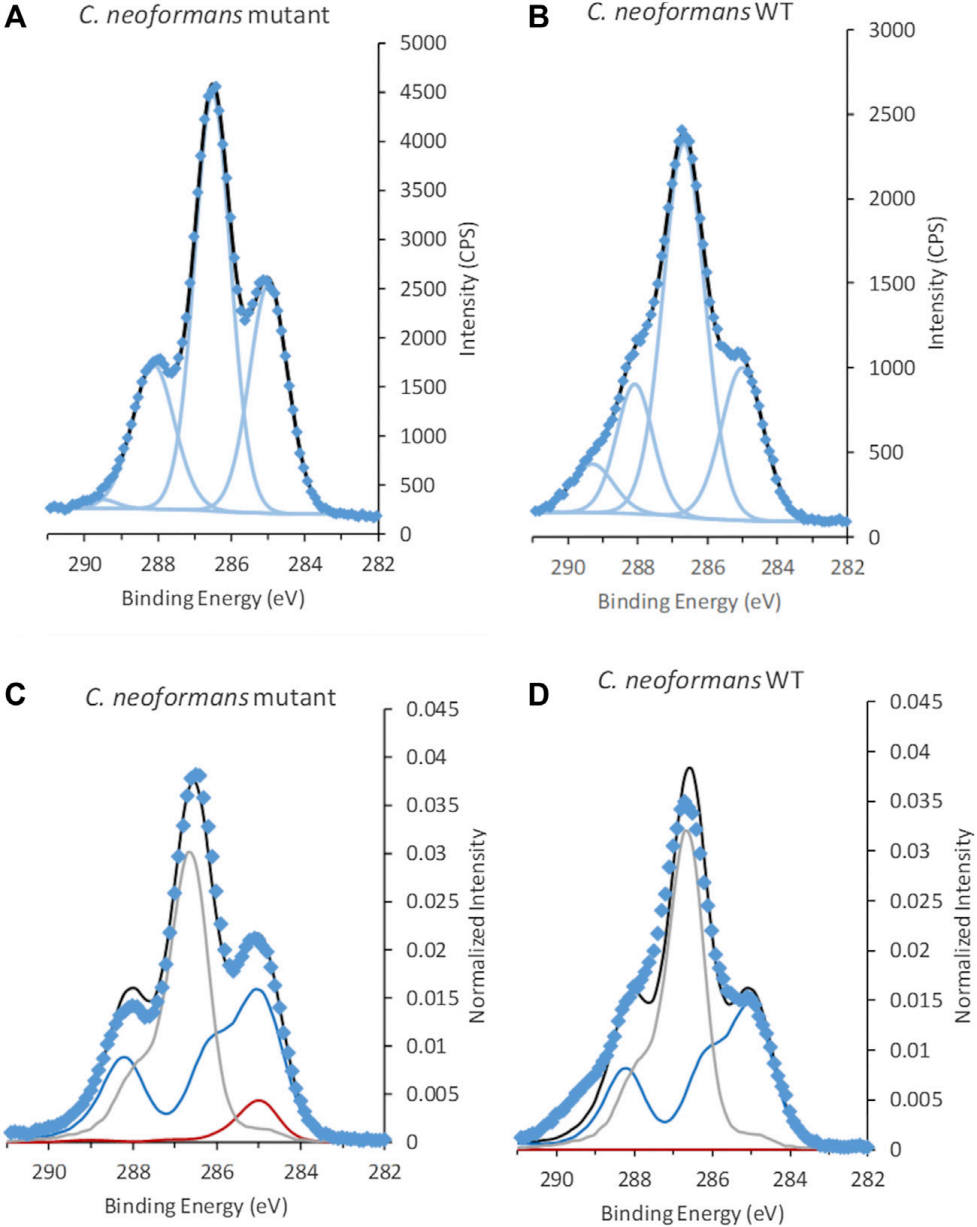
C 1s spectra from *C. neoformans* **(A,C)** mutant without capsule and **(B,D)** wild type (WT) with capsule. Spectra in **(A,B)** are fitted using traditional fitting of Gaussian-Lorentzian (GL30) peak shapes, **(C,D)** fitted using spectral components. Data is represented by blue diamonds, the fit by a solid black line, GL30 peak shapes are represented by light blue lines in figures **(A,B)**, and in **(C,D)** the blue line represents protein-like substance, grey line polysaccharides and red line lipid-like substances.

C 1s spectra acquired from *C. neoformans* cells with and without capsule (mutant) showed a clear difference in peak shape, indicating presence of other types of building blocks in the capsule (Figure 5 and Supplementary Figure S4). Fitting of the C 1s spectra with GL30 peak shapes visualized some differences between the cells, for example, aliphatic C atoms, at 285.0 eV, corresponding to 32 ± 5% of C 1s in the sample without capsule, and 21 ± 3% of C 1s in samples with capsule (Table 3). The peak at 286.6 eV assigned to C-O exhibited similar contributions between the strains and is hypothesized to a large extent originate from polysaccharides. The peak assigned to carboxylate groups at 289.5 eV has higher intensities in data from the WT strain with capsule (Table 3 and Supplementary Table S2). The N/C_tot_ ratio was similar with 0.11 ± 0.03 in the sample without capsule and 0.08 ± 0.03 in the sample with capsule. These results agree well with the expected change in surface composition where a capsule containing polysaccharides with carboxylic groups would lead to an increase in the signal at 289.5 eV but “dilute” signal from aliphatic carbon and peptide C, and decrease the apparent N content.

Interestingly, the *C. neoformans* WT spectra all showed a slight broadening of the peaks giving rise to wider Full Width at Half Maximum (FWHM) when fitted compared to the other samples (Supplementary Table S2). This feature was reproducible (*n* = 5) and did not change with settings of the charge neutralizing system or for samples analyzed “floating,” i.e., does not appear to be a result of differential charging. The peak widths remained also after dehydration of the sample in the sample-introducing chamber (Supplementary Figure S4), thus, indicating that it was not caused by possible formation of a double electric layer at the interface between the capsule and the beta-glucan layer at the cell surface. The significant difference in carboxylic groups between the two *C. neoformans* strains suggested that this broadening may be linked to secondary C 1s chemical shifts of neighboring C atoms. In order to further investigate this, a second fitting strategy was applied to C 1s spectra of the WT strain where an additional component relating to C-COOH was introduced at 285.4 eV (Beamson and Briggs, 1992). In addition, the FWHM for all components in C 1s were restricted to maximum 1.4 eV. Using this fitting approach, a comparably good fit was obtained where the components converged to a FWHM of 1.4, and an approximate 1:1 ratio between the C-COOH and COOH components, at 285.4 and 289.5 eV, was obtained supporting this hypothesis (Supplementary Table S3). Depending on the position of COOH groups (in the hexose ring or elsewhere) such peak “broadening” would appear in the peak at 285.0 eV, at 286.6 eV, or in both components.

The overall substance composition obtained from the spectral model was not significantly different between the two samples, and predicted approximately equal amounts of peptide and polysaccharide (Table 3). This probably relates to the intensity contributions at high BE in C 1s spectra of the WT that are not captured by the model. The capsule is expected to consist of building blocks such as mannose, xylose and glucuronic acid, which would be interpreted by the spectral components as polysaccharide but with unexplained intensity representing the carboxyl group on glucuronic acid (Shchukarev et al., 2020). As the area represented by carboxyl groups are not included in the prediction this would lead to an under-estimation of polysaccharides by the spectral model. Furthermore, the spectral components have a fixed shape and cannot accommodate for changes in peak FWHM. Thus, the deviation between the model and the data may also be relating to the consistent broadening of the peaks of the WT strain, discussed above.

Overall, the analyses of these two species of fungi illustrate that the approach used here, with cryo-XPS and two subsequent data treatment methods, applies well to some fungal cells that do not have surface components differing substantially from those of bacterial cells. For strains such as *C. albicans*, with a surface layer composed of mainly mannan, both data treatments worked well and provided models with god fit to the data. For *C. neoformans* fitting using GL30 peak shapes could to some extent capture the compositional changes expected from literature. However, the data treatment procedure using spectral components showed an under-prediction of polysaccharide due to presence of high levels of carboxylic acid functional groups, which are not well captured by the idealized spectral components. Thus, giving rise to a poor fit of the model to the fungal cells with capsule. This illustrates the limitations of the spectral model for microbial cells with a surface chemistry differing substantially from that of bacterial cells. Consequently, the applicability of the spectral model to fungal cells appears to be species dependent and further method development would be needed to accurately predict the content of polysaccharides with large contributions of, e.g., glucuronic acid (similarly as discussed for nucleic acids above). Irrespective of this, cryo-XPS analyses of all fungal cells gave reproducible data and could visualize the surface chemical changes described in literature when both classical fitting using GL30 peak shapes and the model spectral components were used. But there is room for improvement in the latter data treatment method in order to predict surface composition of microorganisms with surface composition differing substantially from what is commonly present in bacteria.

### Phage Samples

**FIGURE 6:**
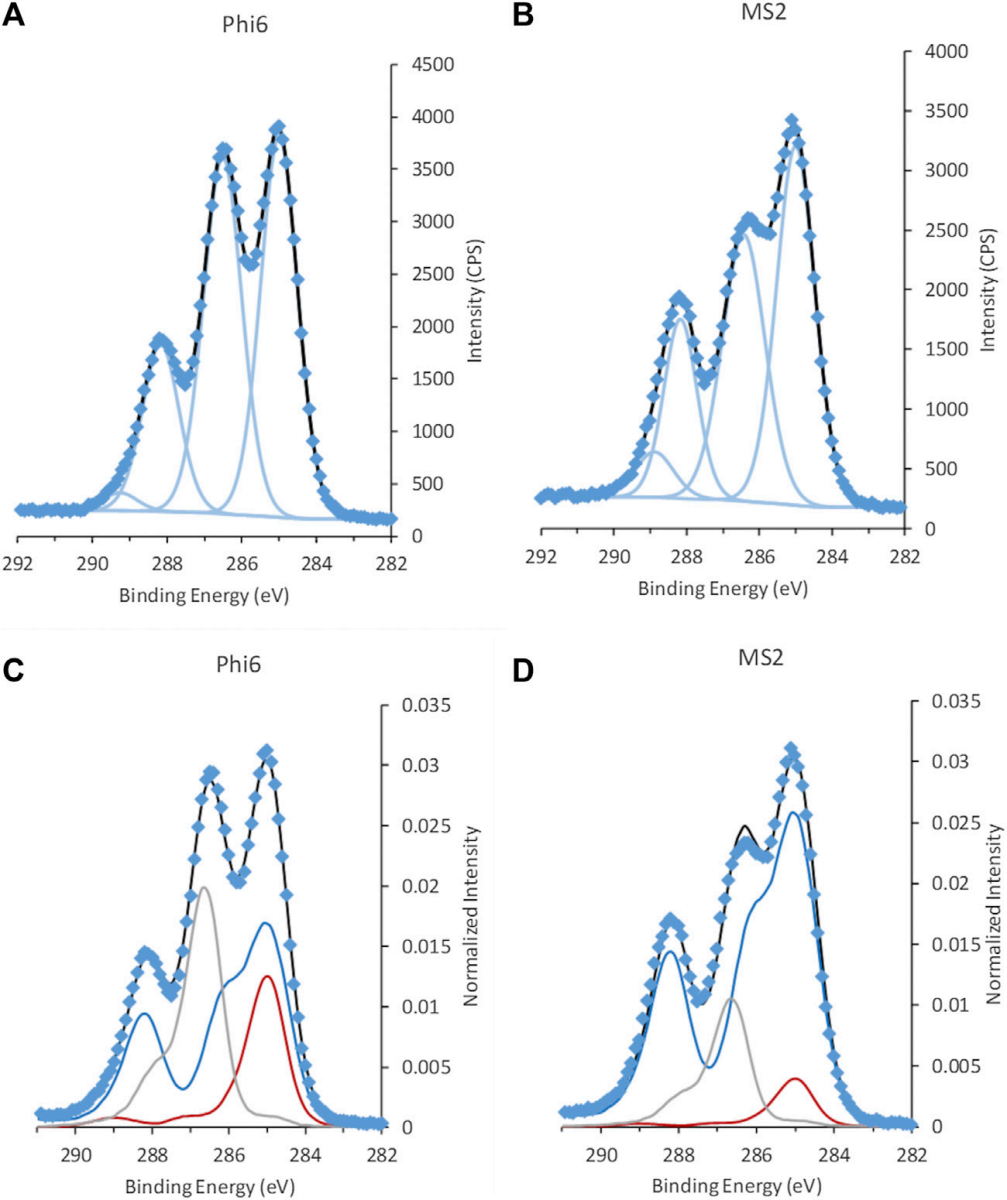
C 1s spectra from the two bacteriophages **(A,C)** Phi6 and **(B,D)** MS2, fitted with GL30 peak shapes **(A,B)** and spectral components **(C,D)**. Data is represented by blue diamonds, the fit by a solid black line, GL30 peak shapes are represented by light blue lines in figures **(A,B)**, and in **(C,D)** the blue line represents protein-like substance, grey line polysaccharides and red line lipid-like substances.

The C 1s spectra of the non-enveloped MS2 and the lipid-enveloped Phi6 phage showed clear differences in intensity at 286.4–286.5 eV, assigned to C with a single bond to O or N (Figure 6 and reproducibility in Supplementary Figure S5; Supplementary Table S4). The high-resolution spectra of most other elements that are present in the samples, namely Na, O, Cl, P, and S, were similar in shape and quantity between the two phages (Supplemental Table S4). The exception was N with the ratio N/C = 0.14 ± 0.01 for Phi6 and N/C = 0.24 ± 0.01 for MS2. As the phages were suspended in PBS, the spectra from Na, O, Cl, and P are expected to have significant contributions from the solvent, and were not examined further.

When fitted with spectral components, the difference between the two phages became even more evident (Figure 6; Table 3). The difference in surface content related to more lipid (24 ± 5% of total C atoms) and polysaccharide (28 ± 1% of total C atoms) at the surface of Phi6, compared to the surface of MS2 mainly consisting of protein (81 ± 2% of total C atoms). This is in line with the substance composition described in the literature: In Phi6, the lipids are reported to make up 20 wt%, proteins 70 wt%, and nucleic acids 10 wt% of the particle (King et al., 2011). MS2 has been reported to have 39 wt% nucleic acids and the rest of the weight from proteins (King et al., 2011).

The surface structures for Phi6 consist of the spikes from glycosylated proteins and the lipid envelope surrounding the protein capsid. For the non-enveloped MS2, the surface is formed by the protein capsid. This corresponds well with the data obtained with respect to both N/C ratio and composition of the spectral components. The fit of the spectral model to the phage data was good (Figure 6). The type of deviations observed for the RNA standard were not seen for the phage samples (Figure 3 and Supplementary Figure S5), indicating that nucleic acids were present below the information depth (<10 nm) of the XPS. This agrees well with our predictions based on the size and morphology of the phages, i.e., a diameter of 85 nm for Phi6 and of 25–27 nm for MS2 (Watts et al., 2020). The protein shell thickness in the virus capsid of MS2 was analyzed to be around 6 nm from small angle X-ray scattering (Watts et al., 2020). Based on the XPS data and the information on virus size and morphology, we hypothesize that cryo-XPS mostly allows for analysis of the viral capsid for non-enveloped viruses and not the RNA core. For enveloped viruses the lipid envelope, the capsid and surface structures can be analyzed.

## Conclusion

The surface chemical composition of both fungal cells and viral particles can be well characterized using cryo-XPS in combination with data-treatment approaches previously developed for bacterial cells. Thus, clearly illustrating that the surface chemistry of microorganisms with very different structure and life forms can be monitored intact and hydrated using cryo-XPS, with a minimal of sample preparation.

Surface layers of fungal cells are highly species-dependent and dynamically responsive to environmental cues. Thus, they represent systems where cryo-XPS has a potential to bring an increased understanding of processes at the near surface of the cells. XPS data from *C. albicans,* with its outermost layers consisting of mannan, was well fitted with both data treatment methods giving results agreeing with literature. The surface chemical composition of *C. neoformans,* with its large amounts of capsular carboxylic acid functional groups, was challenging for the spectral model and predicted an overall lower polysaccharide content than expected. These difference originated from the model not capturing intensity linked to carboxylic groups, e.g. in glucuronic acid. This is similar to what has been described for microalgae, glucuronic acid and glucosamine (Shchukarev et al., 2020). These systematic differences suggests that the Umeå method could be improved by developing spectral components from a large dataset including microorganisms with variation in these building blocks relating to the eukaryote cell wall. A large dataset would allow for development of a new collection of spectral components (based on multivariate curve resolution) that could predict the presence of substances foreign to bacterial cells. However, until such a model is developed, the traditional fitting using GL30 peak shapes can be used. Furthermore, the presence of capsular building blocks will be visible as systematic deviations between the fit from spectral components and data. In combination with the fitting using GL30 components, capsular presence (or absence) could be observed here in the data from fungal cells and gave results in line with what has previously been reported.

The data from bacteriophages, indicated that cryo-XPS probes only the outer layers of the viruses. These layers are represented by the protein capsid, the peplomers and the lipid envelope for enveloped viruses, as well as the protein capsid for non-enveloped ones. The surface chemical composition obtained for the viruses agree well with literature data. The data could be fitted with both the GL30 peak shapes and the spectral components constructed for the bacterial cell envelope. Overall, these results demonstrate that cryo-XPS is well applicable to characterize the surface chemical composition of viruses. The good fit between the spectral model and the experimental data furthermore indicate that, e.g., nucleic acids are present below the information depth of XPS, at the center of the viral particle.

## Data Avalability Statement

The datasets generated for this study in the form of reference XPS spectra of building blocks can be found online at Spectroscopy Hub [spectroscopyhub.com].
