# Supplementary material for: Applying Cryo-X-ray Photoelectron Spectroscopy to Study the Surface Chemical Composition of Fungi and Viruses
Source: Front Chem. 2021 May 28;9:666853. doi: 10.3389/fchem.2021.666853 (PMC8194281; doi:10.3389/fchem.2021.666853)
Supplement: Supplementary file 1 [file DataSheet1.PDF]

Supplementary Material

Supplementary Material

1 Supplementary Figures and Tables

1.1 Supplementary Figures

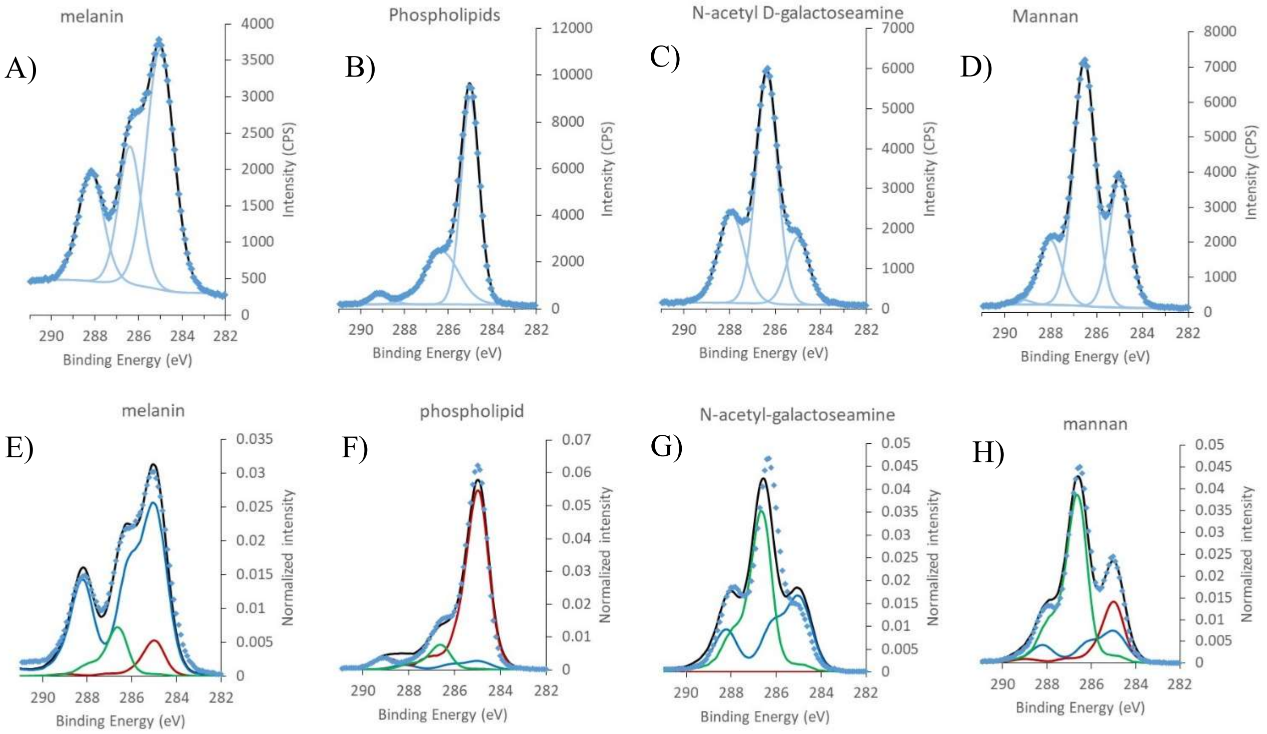

Figure S1. C1s spectra of reference samples fitted with Gaussian-Lorentzian (GL30) peaks (CasaXPS) as well as with spectral components (MATLAB). Data is represented by blue diamonds, the fit by a solid black line. GL30 peak shapes are represented by light blue lines in A-D. The blue line represents protein-like substance, green line polysaccharides and red line lipid-like substances in figures E - H. A & E) melanin, B & F) phospholipids, C & G) N-acetyl galatosamine D&H) mannan

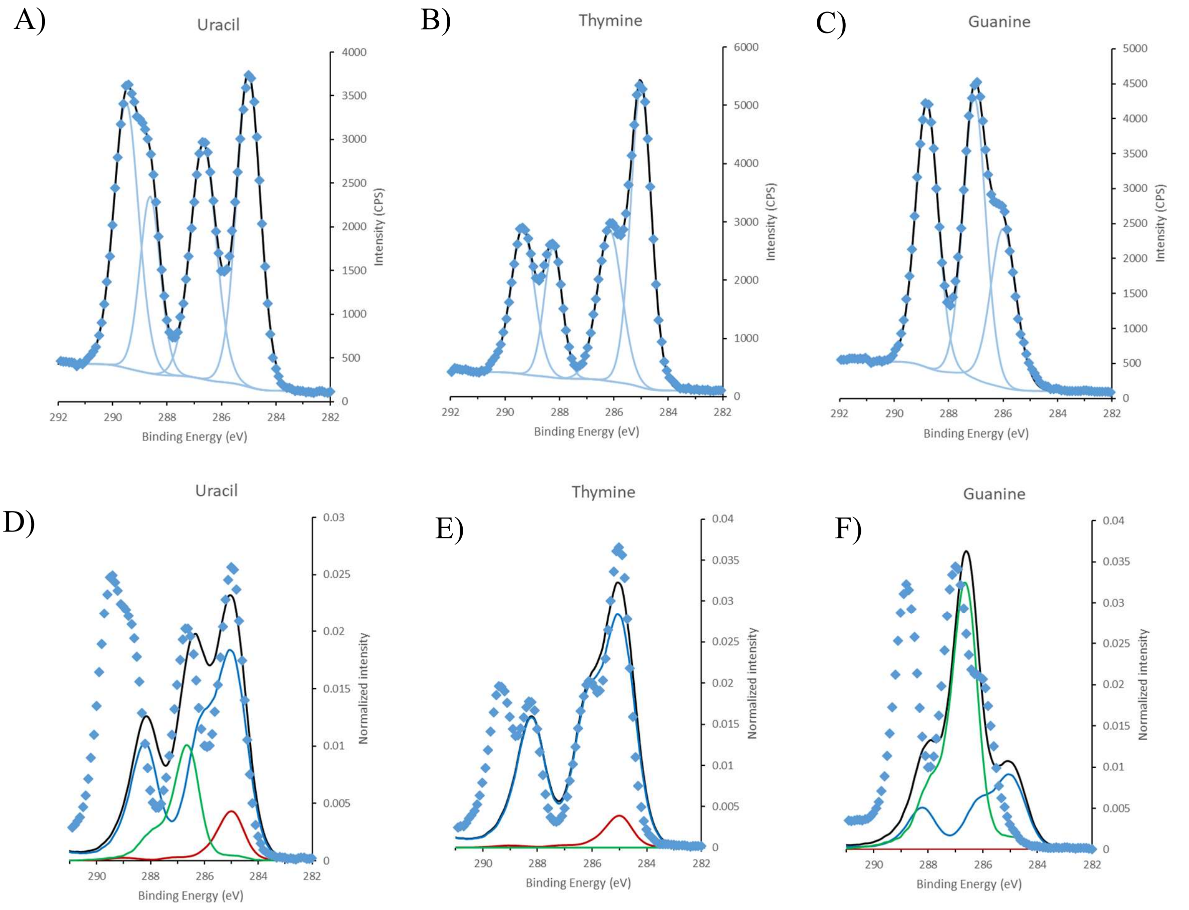
A)
Uracil
B)
Thymine
C)
Guanine
Intensity (CPS)
Binding Energy (eV)
D)
Uracil
E)
Thymine
F)
Guanine
Normalized intensity
Binding Energy (eV)

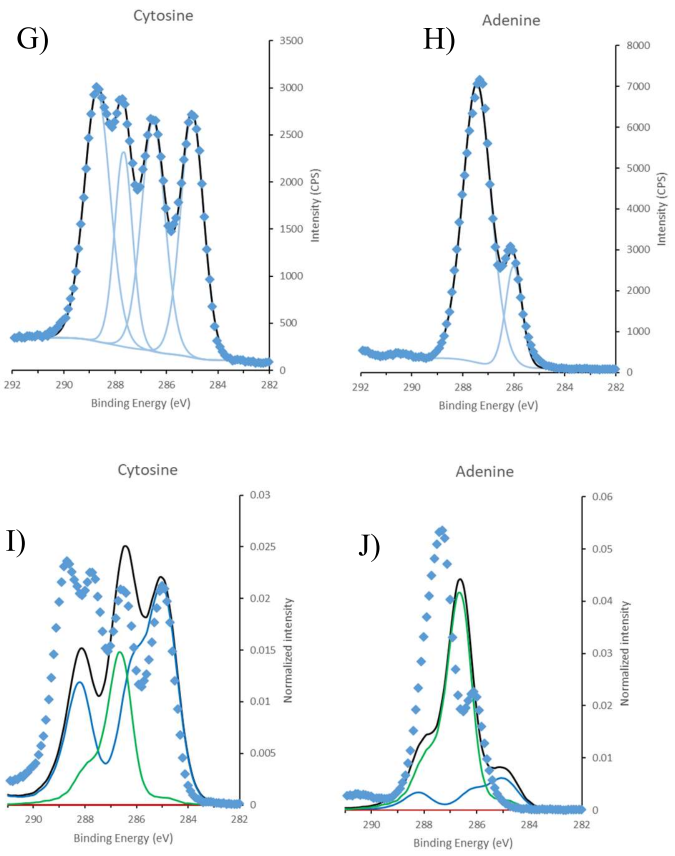

Figure S2. C1s spectra of bases in DNA and RNA fitted with Gaussian-Lorentzian (GL30) peaks (CasaXPS) as well as with spectral components (MATLAB). Data is represented by blue diamonds, the fit by a solid black line. GL30 peak shapes are represented by light blue lines in figures A-C and G-H. The blue line represents protein-like substance, green line polysaccharides and red line lipid-like substances in figures D-F and I-J. A & D) uracil, B & E) thymine, C & F) guanine, G & I) cytosine, H & J) adenine.

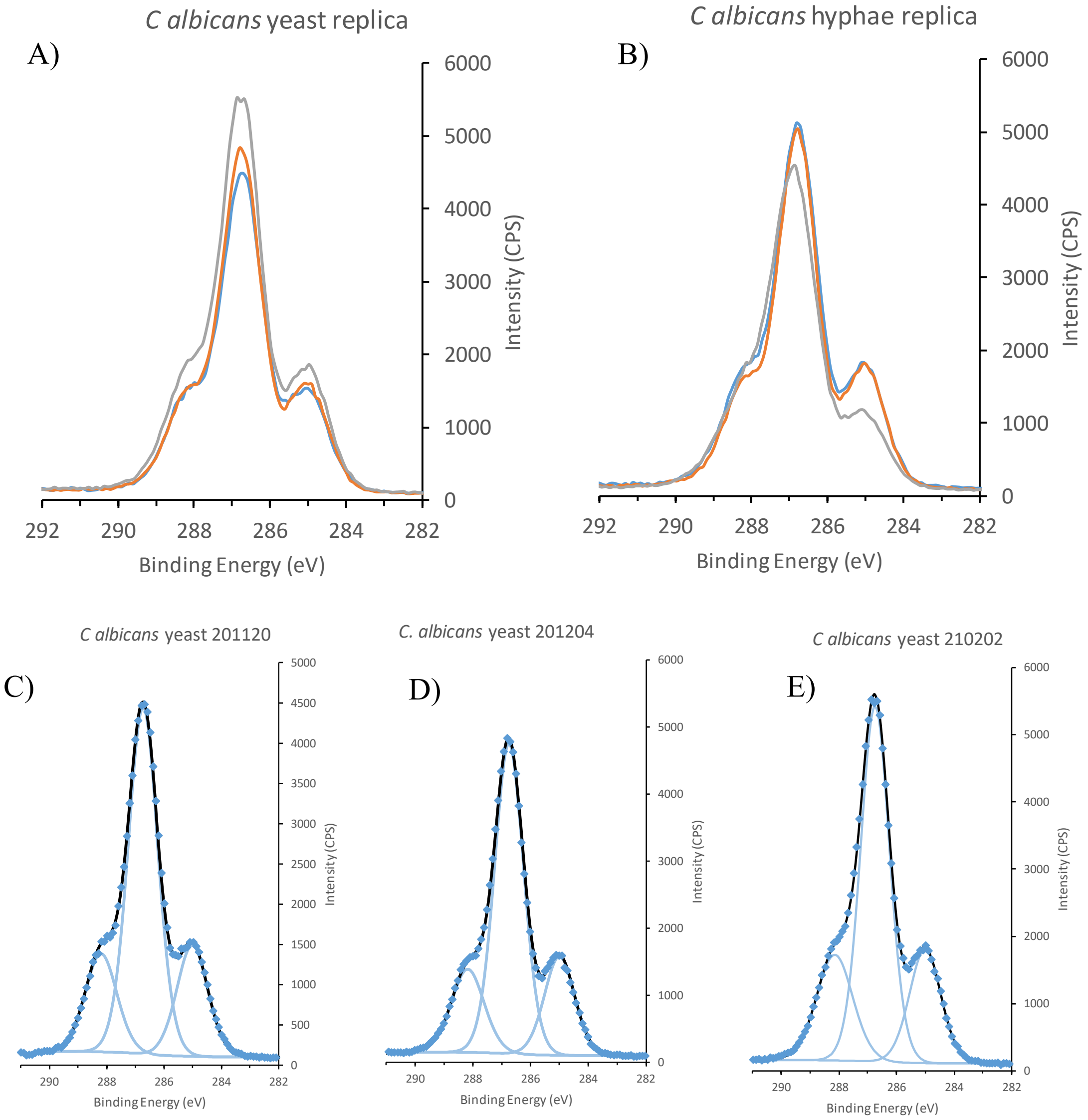
C albicans yeast replica
A)
6000
5000
4000
3000
2000
1000
0
Intensity (CPS)
292 290 288 286 284 282
Binding Energy (eV)
C albicans hyphae replica
B)
6000
5000
4000
3000
2000
1000
0
Intensity (CPS)
292 290 288 286 284 282
Binding Energy (eV)
C albicans yeast 201120
C)
5000 4500 4000 3500 3000 2500 2000 1500 1000 500 0
Intensity (CPS)
290 288 286 284 282
Binding Energy (eV)
C. albicans yeast 201204
D)
6000 5000 4000 3000 2000 1000 0
Intensity (CPS)
290 288 286 284 282
Binding Energy (eV)
C albicans yeast 210202
E)
6000 5000 4000 3000 2000 1000 0
Intensity (CPS)
290 288 286 284 282
Binding Energy (eV)

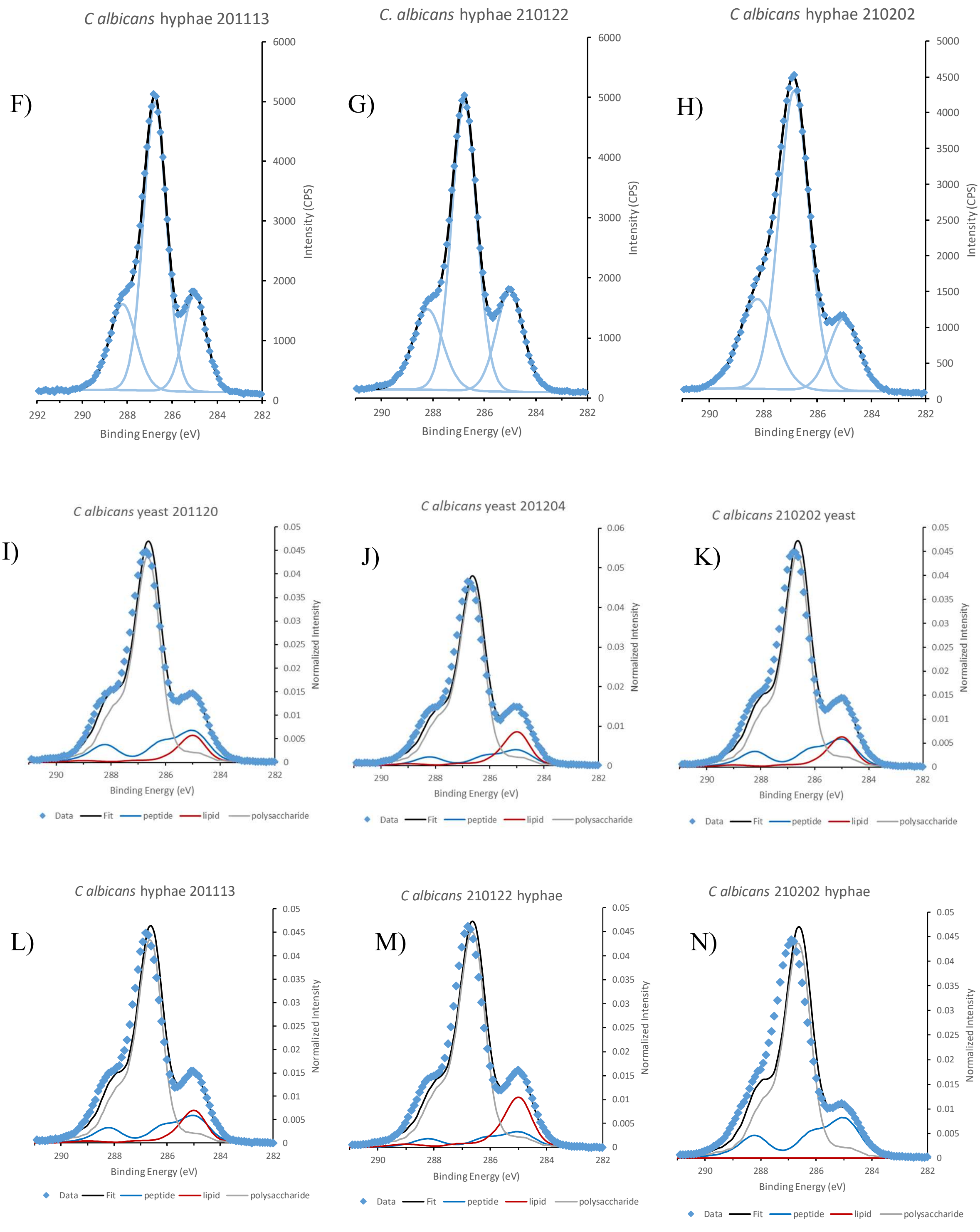

Figure S3. A-B) C1s spectra of all replica of *C. albicans* with fitting of C-H) GL30 peak shapes and I-N) spectral components. Data is represented by blue diamonds, the fit by a solid black line. In C-H) the GL30 peak shapes are presented in blue, and in I-N) the blue line represents protein-like substance, grey line polysaccharides and red line lipid-like substances.

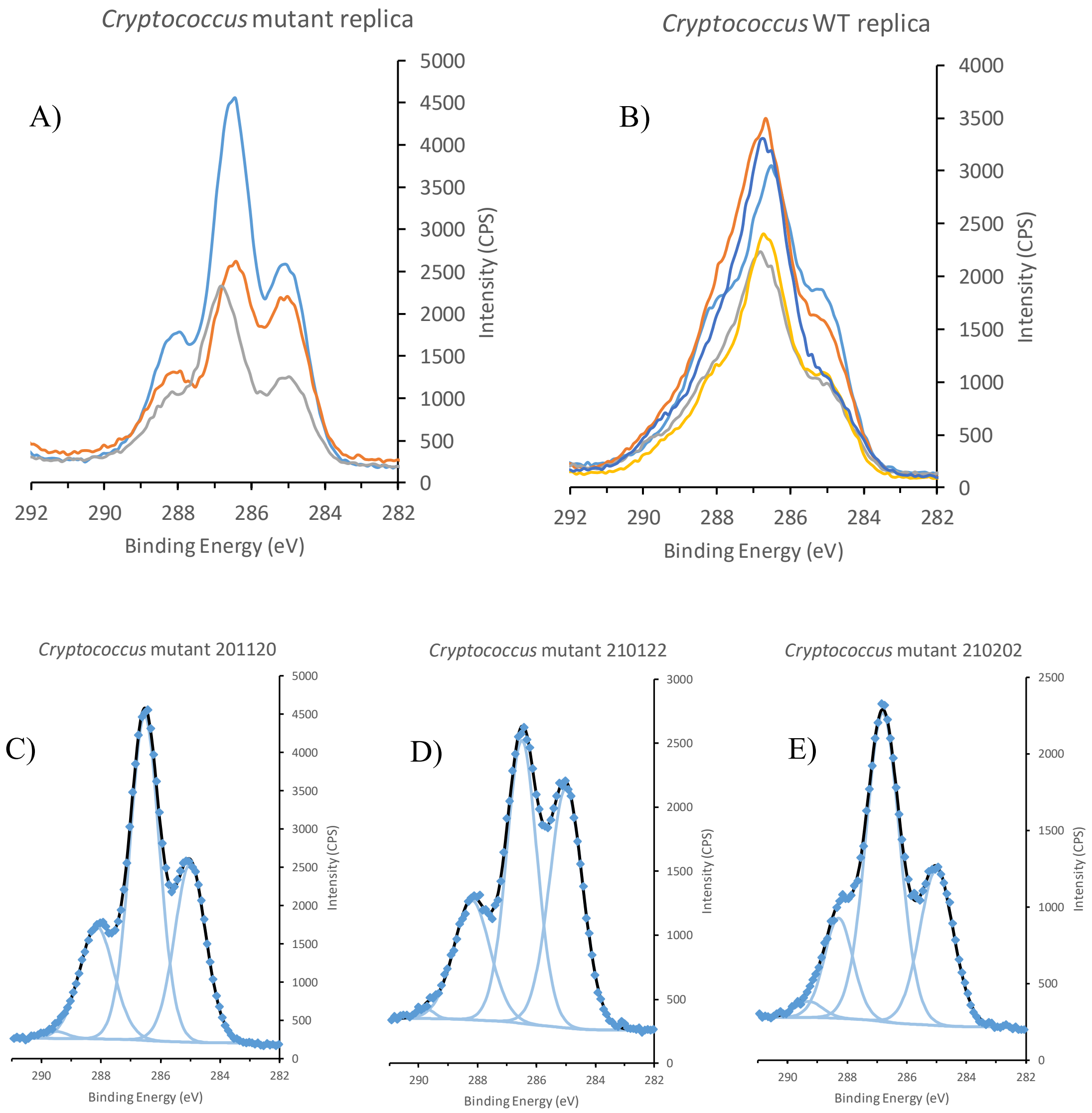

A)
Cryptococcus mutant replica
B)
Cryptococcus WT replica
Intensity (CPS)
Binding Energy (eV)
C)
Cryptococcus mutant 201120
D)
Cryptococcus mutant 210122
E)
Cryptococcus mutant 210202

F)

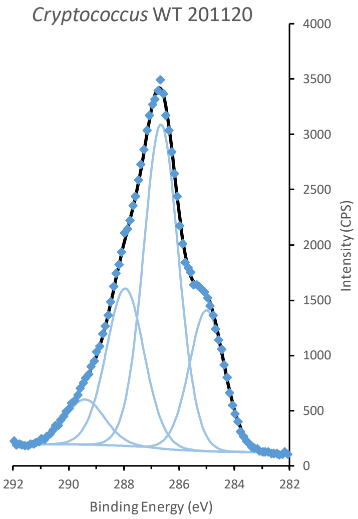
Cryptococcus WT 201120
Intensity (CPS)
Binding Energy (eV)
0
500
1000
1500
2000
2500
3000
3500
4000
292
290
288
286
284
282

G)

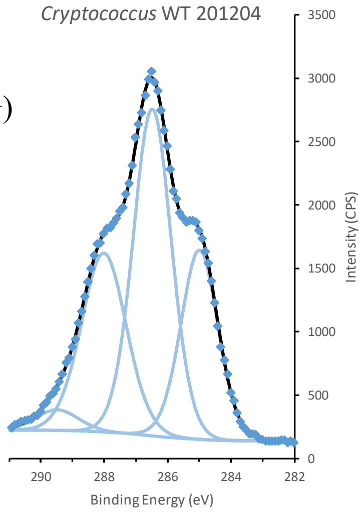
Cryptococcus WT 201204
Intensity (CPS)
Binding Energy (eV)
0
500
1000
1500
2000
2500
3000
3500
290
288
286
284
282

H)

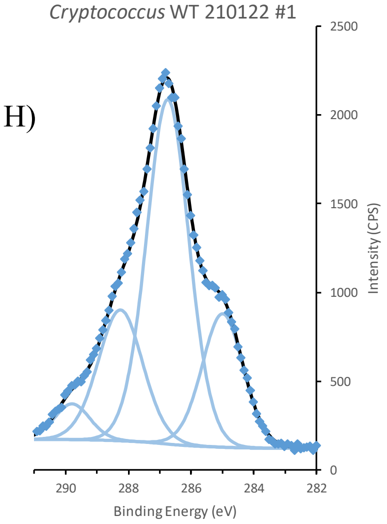
Cryptococcus WT 210122 #1
Intensity (CPS)
Binding Energy (eV)
0
500
1000
1500
2000
2500
290
288
286
284
282

I)

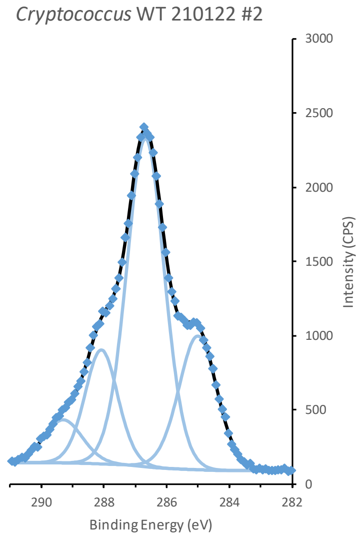
Cryptococcus WT 210122 #2
Intensity (CPS)
Binding Energy (eV)
0
500
1000
1500
2000
2500
3000
290
288
286
284
282

J)

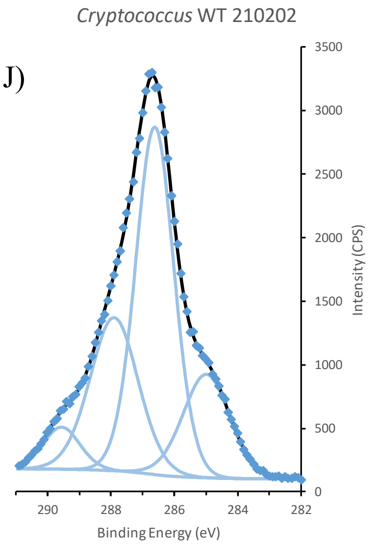
Cryptococcus WT 210202
Intensity (CPS)
Binding Energy (eV)
0
500
1000
1500
2000
2500
3000
3500
290
288
286
284
282

K)

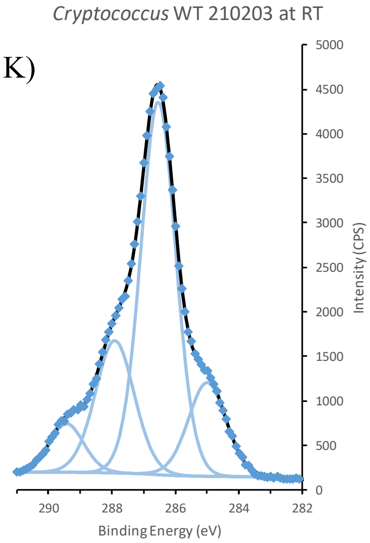
Cryptococcus WT 210203 at RT
Intensity (CPS)
Binding Energy (eV)
0
500
1000
1500
2000
2500
3000
3500
4000
4500
5000
290
288
286
284
282

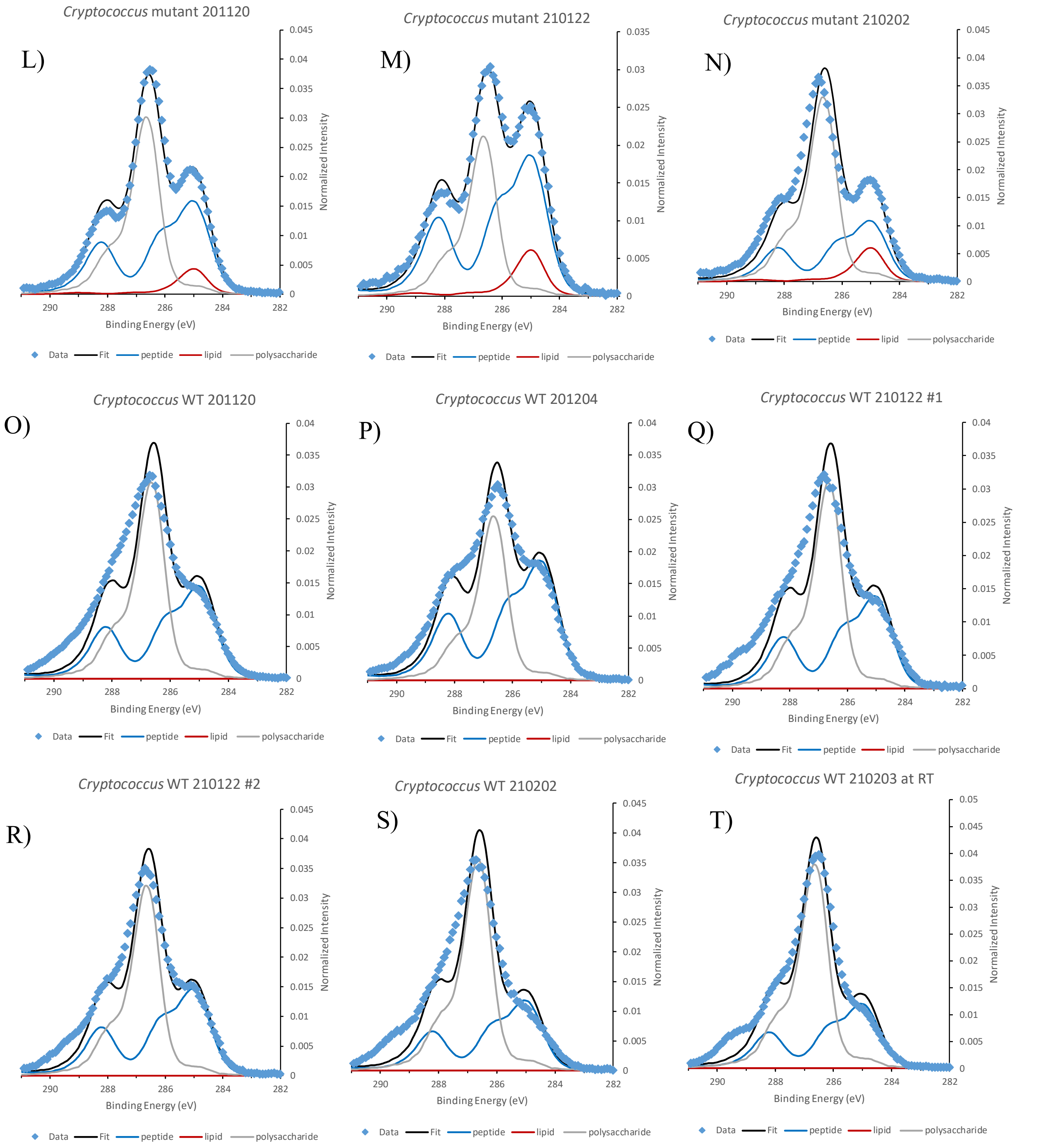

Figure S4. A-B) C1s spectra of all replica of *C. neoformans* with fitting of C-K) GL30 peak shapes and L-T) spectral components, Data is represented by blue diamonds, the fit by a solid black line. In C-K) the blue lines represent the GL30 peak shapes, in L-T) the blue line represents protein-like substance, grey line polysaccharides and red line lipid-like substances.

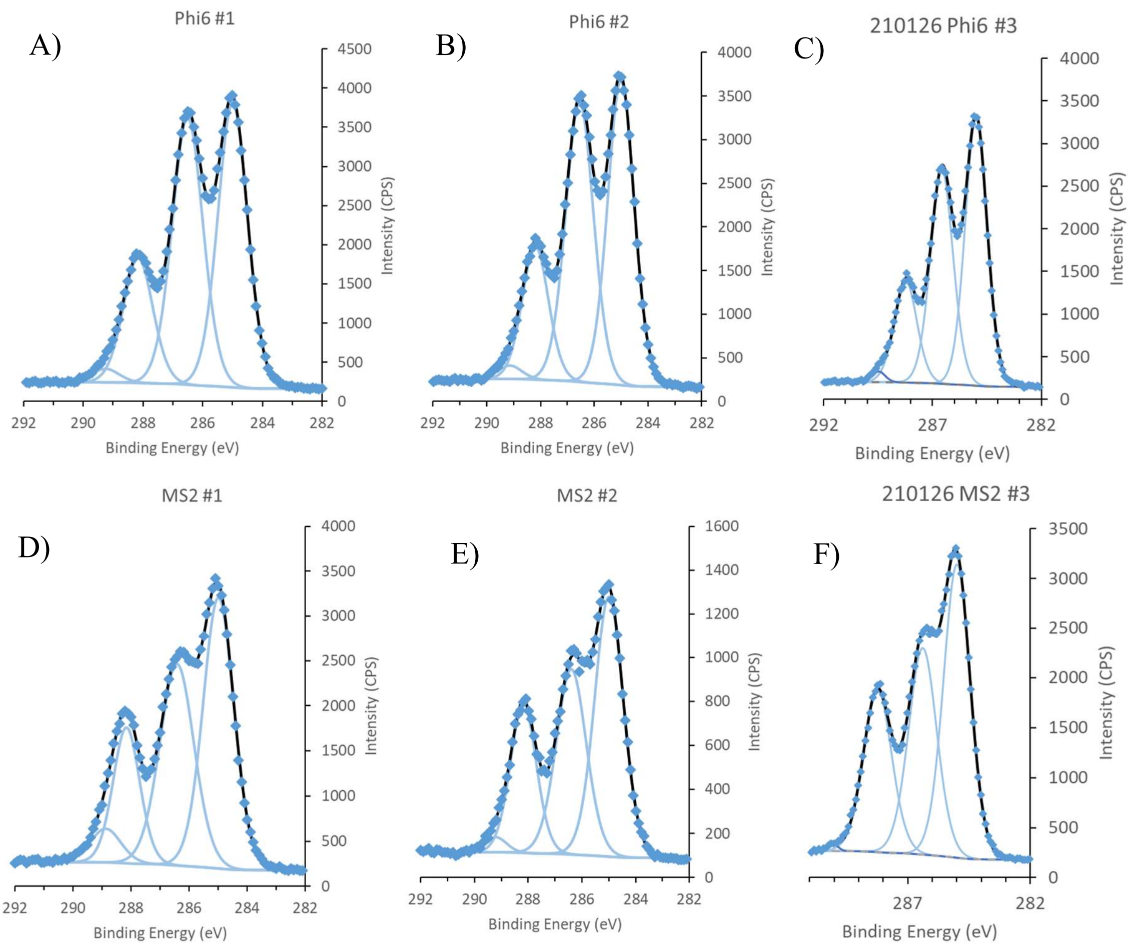
A)
Phi6 #1
B)
Phi6 #2
C)
210126 Phi6 #3
D)
MS2 #1
E)
MS2 #2
F)
210126 MS2 #3
Binding Energy (eV)
Intensity (CPS)

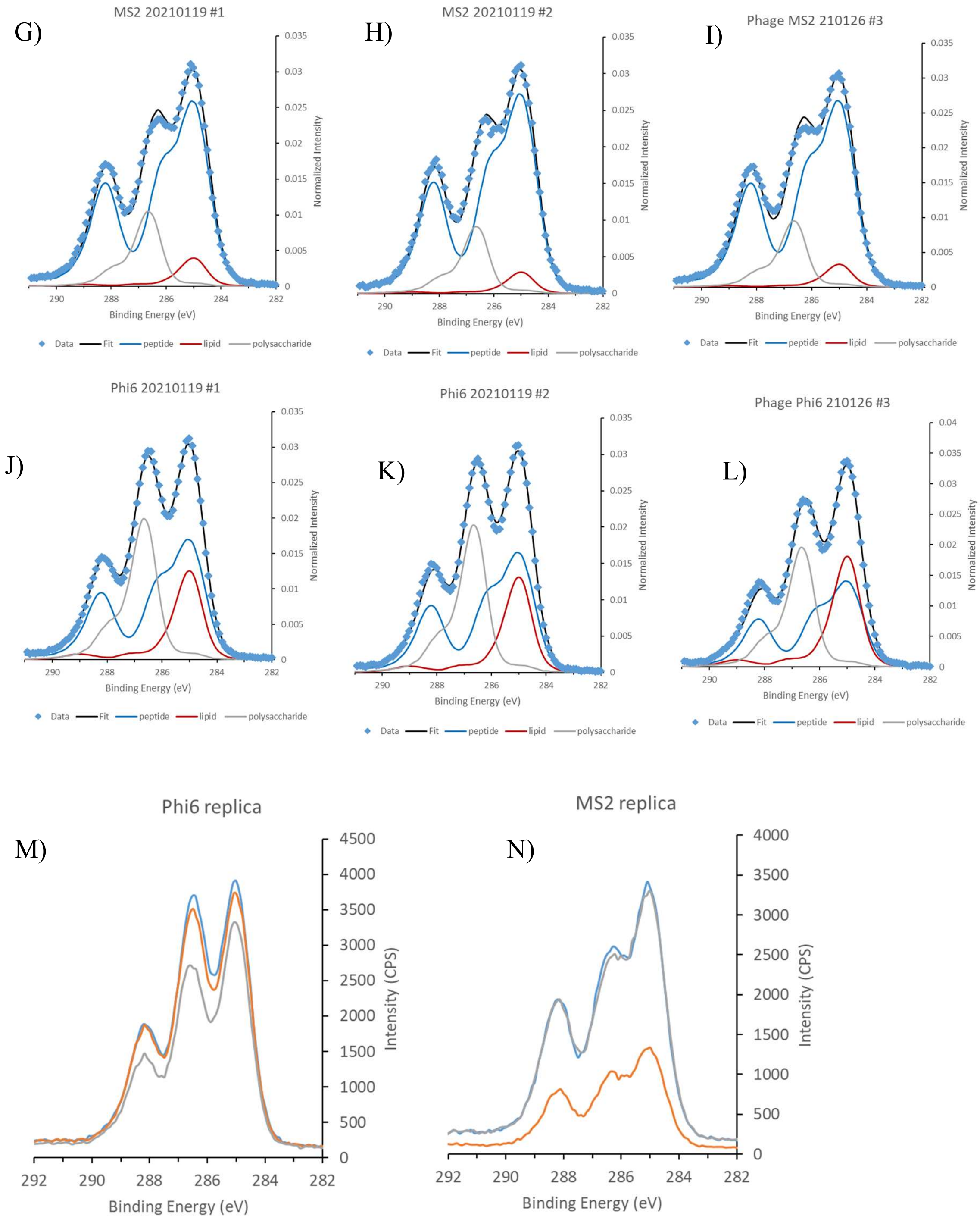

Figure S5. C1s spectra of all replica of the two phages Phi6 and MS2 with fitting, Data is represented by blue diamonds, the fit by a solid black line. Fit in A-F) are represented of GL30 peak shapes in blue, Shirley background in grey, G—L) fit of spectral components; the blue line represents protein-like substance, grey line polysaccharides and red line lipid-like substances, M-N) spectral overlay of raw data.

1.2 Supplementary Tables

Table S1. XPS data relating to fitting of spectra from reference substances using GL(30) peak shapes and CasaXPS

**Fungal references**<br><br>**Binding Energy position (eV)**	**Na 1s**	**O1 1s**	**O2 1s**	**O sum**	**N1 1s**	**N2 1s**	**N sum**	**C1 1s**	**C2 1s**	**C3 1s**	**C4 1s**	**C sum**	**Cl $2p_{3/2}$ or Cl sum**	
melanin@	1072.0@	531.5	532.9		400.1			285.0	286.4	288.2			**197.8**	**199.4@**
mannan	1071.8	531.4	533.0		400.1	401.9		285.0	286.6	288.0	289.2			
d-galactosamine HCl			533.1		399.8	402.0		285.0	286.7	288.3				**198.2**
n-acetyl-d-galactosamine		532.4	532.7		399.8	400.1		285.0	286.4	287.9				
**FWHM**														
melanin@	2.0	1.6	1.5		1.4			1.5	1.2	1.4			**1.2**	**1.8**
mannan	1.6	1.2	1.4		1.4	1.8		1.1	1.1	1.2	0.9			
d-galactosamine HCl			1.4		1.3	1.4		1.1	1.1	1.2				**1.0**
n-acetyl-d-galactosamine		1.9	1.1		1.0	1.4		1.1	1.1	1.3				
**at % concentration**														
melanin	2.5	14.6	4.6	19.3	11.7		11.7	33.9	16.0	14.0		63.9		2.7
mannan	0.4	2.7	29.7	32.4	2.3	0.2	2.5	18.3	35.8	10.0	0.5	64.7		
d-galactosamine HCl			34.7	34.7	0.7	6.7	7.4	4.3	37.8	9.9		52.0		5.9
n-acetyl-d-galactosamine		24.2	12.2	36.4	3.1	3.2	6.4	9.9	32.0	15.3		57.2		
**ratio to total C**														
melanin	0.038			0.302			0.184	0.530	0.250	0.220				0.042
mannan	0.006			0.501			0.039	0.284	0.553	0.155	0.008			
d-galactosamine HCl				0.668			0.142	0.082	0.727	0.191				0.113
n-acetyl-d-galactosamine				0.636			0.111	0.173	0.559	0.267				

@=suspected NaCl impurity in the standard

Virus references	Na1 1s	Na2 1s	Na sum	O1 1s	O2 1s	O sum	N1 1s	N2 1s	N sum	C1 1s	C2 1s	C3 1s	C4 1s	C sum	P $2p_{3/2}$ or Ptot
**Binding Energy position (eV)**															
cholesterol					532.8					285.0	285.7				
phospholipids				530.9	532.7		400.3	402.6		285.0	286.3		289.1		133.5
RNA				531.5	533.1		399.7	401.0		285.0	286.8	288.2	289.7		133.9
DNA	1071.4	1073.0		531.1	532.8		399.1	400.5		285.0	286.6	287.9	289.2		133.4
**FWHM**															
cholesterol					1.3					0.9	1.5				
phospholipids				1.0	1.9		2.4	1.3		1.0	1.9		1.2		1.2
RNA				1.5	1.5		1.5	1.6		1.2	1.3	1.6	1.2		1.5
DNA	1.5	1.5		1.4	1.5		1.3	1.6		1.2	1.4	1.5	1.2		1.3
**at % concentration**															
cholesterol					3.2	3.2				78.7	18.1			96.8	
phospholipids				1.7	15.6	17.3	0.5	0.5	1.1	51.5	25.5		3.2	80.3	1.3
RNA				9.8	22.1	31.9	5.2	9.3	14.5	8.5	27.1	12.3	1.8	49.7	3.9
DNA	4.1	0.2	4.3	11.4	13.7	25.0	7.0	8.1	15.1	18.1	22.6	9.8	1.5	52.0	3.6
**ratio to total C**															
cholesterol						0.033				0.813	0.187				
phospholipids						0.216			0.014	0.642	0.318		0.040		0.016
RNA						0.642			0.292	0.170	0.546	0.247	0.037		0.079
DNA			0.082			0.481			0.290	0.348	0.435	0.189	0.028		0.069

Nucleobases	C1 1s	C2 1s	C3 1s	C4 1s	C5 1s	C sum	N1 1s	N2 1s	N sum	O1 1s	O2 1s	O sum	Na 1s
**Binding Energy Position (eV)**													
uracil	285.0	286.7		288.6	289.5		400.8			531.8	532.4		
thymine	285.0	286.2		288.2	289.4		400.6			531.7			
cytosine	285.0	286.5	287.7	288.7			399.9	398.8		531.3	531.9		1071.9
guanine§		286.0§	287.1	288.8			399.6	401.0			532.1		
adenin§		286.0§	287.4				399.8	401.0			533.2		
**FWHM**													
uracil	1.0	1.1		0.9	1.1		1.2			1.1	1.5		
thymine	0.9	1.1		0.8	1.0		1.1			1.3			
cytosine	1.1	1.1	0.8	1.2			1.6	0.9		1.1	1.5		1.6
guanine		1.1	1.0	1.0			1.1	1.4			1.2		
adenin		0.8	1.3				1.0	1.4			2.6		
**at % concentration**													
uracil	16.9	13.8		8.2	15.0	53.9	24.0		24.0	14.7	7.4	22.1	
thymine	22.9	13.6		8.6	12.5	57.6	22.6		22.6	19.8		19.8	
cytosine	13.7	13.3	8.5	16.0		51.5	30.9	5.9	36.8	8.0	3.4	11.4	0.3
guanine		11.2	17.5	16.5		45.2	23.2	23.4	46.6		8.2	8.2	
adenin		8.7	41.2			50.0	35.0	14.2	49.2		0.8	0.8	
**ratio to total C**													
uracil	0.314	0.255		0.152	0.278			0.445			0.410		
thymine	0.397	0.237		0.150	0.217			0.393			0.343		
cytosine	0.267	0.258	0.166	0.310				0.713			0.222		0.006
guanine	0.000	0.248	0.387	0.364				1.031			0.181		
adenin		0.175	0.825					0.985			0.017		

§=Binding energy scale calibrated to 286.0 eV relating to **C**-N [ref: Beamson, G., and Briggs, D. (1992) High resolution XPS of organic polymers: the Scienta ESCA300 database, Wiley, New York]

Table S2. XPS data from fungal replica including averages and standard deviations (please note that the minor elements were not acquired for all samples due to their very low quantity at the surface).

Binding Energy position (eV)	Na 1s	O1 1s	O2 1s	N1 1s	N2 1s	C1 1s	C2 1s	C3 1s	C4 1s	K 2p	K 2p	Cl 2p	Cl 2p	S 2p	P 2p
201120_*C. albicans*_yeast	1071.6	533.1	531.4	400.1	401.5	285.0	286.7	288.2							134.2
201204_*C. albicans*_yeast	1071.7	533.1	531.4	400.2	402.0	285.0	286.8	288.2		296.2	293.1				134.3
210202_*C. albicans*_yeast	1071.9	533.1	531.4	400.2	401.6	285.0	286.7	288.1						164.0	134.2
average C albicans yeast	*1071.7*	*533.1*	*531.4*	*400.1*	*401.7*	*285.0*	*286.7*	*288.2*		*296.2*	*293.1*			*164.0*	*134.2*
st dev	*0.1*	*0.0*	*0.0*	*0.0*	*0.3*		*0.0*	*0.0*							*0.1*
201113_2_*C. albicans* hyphae	1071.7	533.2	531.4	400.1	401.7	285.0	286.8	288.2							134.2
210122_*C. albicans* hyphae	1071.9	533.2	531.4	400.2	401.9	285.0	286.8	288.2						164.4	134.3
210202_*C. albicans* hyphae	1072.0	533.3	531.3	400.2		285.0	286.9	288.2							134.3
average C albicans hyphae	*1071.9*	*533.2*	*531.4*	*400.2*	*401.8*	*285.0*	*286.8*	*288.2*						*164.4*	*134.3*
st dev	*0.1*	*0.0*	*0.1*	*0.1*	*0.2*		*0.1*	*0.0*							*0.0*
201120_*C. neoformans* mutant	1071.4	532.8	531.4	400.0	401.6	285.0	286.5	288.1	289.5	293.1	295.9	198.1	199.7	163.9	133.7
210122_*C. neoformans* mutant	1071.3	533.0	531.4	400.0	401.6	285.0	286.5	288.2	289.7	293.0	295.8	198.2	199.8		133.6
210202_*C. neoformans* mutant	1071.5	533.2	531.5	400.1	401.9	285.0	286.8	288.3	289.3	293.1	295.9	198.3	199.9		133.9
average C. neoformans mutant	*1071.4*	*533.0*	*531.4*	*400.0*	*401.7*	*285.0*	*286.6*	*288.2*	*289.5*	*293.0*	*295.9*	*198.2*	*199.8*	*163.9*	*133.7*
st dev	*0.1*	*0.2*	*0.1*	*0.0*	*0.2*		*0.2*	*0.1*	*0.2*	*0.0*	*0.1*	*0.1*	*0.1*		*0.1*
201120_*C. neoformans* WT	1071.6	533.1	531.2	400.0	401.7	285.0	286.7	287.9	289.4	293.2	296.2	198.1	199.8		133.8
201204_*C. neoformans* WT	1071.4	533.3	531.7	400.1	401.7	285.0	286.5	288.0	289.5	293.0	296.1	198.0	199.4		133.8
210122_*C. neoformans* WT	1071.5	533.3	531.2	400.0	401.7	285.0	286.8	288.3	289.8	293.1	296.0	198.2	199.8		133.7
210122_*C. neoformans* WT	1071.5	533.2	531.2	400.0	401.6	285.0	286.7	288.1	289.3	293.0	296.0	198.1	199.7		133.7
210202_*C. neoformans* WT	1071.7	533.1	531.0	399.9	401.8	285.0	286.6	287.9	289.6			198.0	199.4		
average C. neoformans WT	*1071.5*	*533.2*	*531.2*	*400.0*	*401.7*	*285.0*	*286.6*	*288.0*	*289.5*	*293.1*	*296.1*	*198.1*	*199.6*		*133.8*
st dev	*0.1*	*0.1*	*0.2*	*0.1*	*0.1*		*0.1*	*0.1*	*0.2*	*0.1*	*0.1*	*0.1*	*0.2*		*0.1*

FWHM (eV)	Na 1s	O1 1s	O2 1s	N1 1s	N2 1s	C1 1s	C2 1s	C3 1s	C4 1s	K 2p	K 2p	Cl 2p	Cl 2p	S 2p*	P 2p
201120_*C. albicans*_yeast	1.3	1.5	1.1	1.3	1.6	1.3	1.2	1.3							1.8
201204_*C. albicans*_yeast	1.3	1.5	1.1	1.3	1.2	1.2	1.2	1.3		1.4	1.0				1.8
210202_*C. albicans*_yeast	1.9	1.5	1.1	1.3	1.6	1.3	1.2	1.5						1.4	1.8
average C albicans yeast	*1.5*	*1.5*	*1.1*	*1.3*	*1.5*	*1.3*	*1.2*	*1.4*							*1.8*
st dev	*0.3*	*0.0*	*0.0*	*0.0*	*0.2*	*0.0*	*0.0*	*0.1*							*0.0*

201113_2_*C. albicans* hyphae	1.5	1.6	1.1	1.3	1.6	1.2	1.2	1.4							1.8
210122_*C. albicans* hyphae	1.4	1.5	1.1	1.3	1.4	1.3	1.1	1.4						2.4	1.8
210202_*C. albicans* hyphae	1.6	1.6	1.2	1.6		1.3	1.3	1.6							1.8
average C albicans hyphae	*1.5*	*1.6*	*1.1*	*1.4*	*1.5*	*1.3*	*1.2*	*1.5*						*2.4*	*1.8*
st dev	*0.1*	*0.1*	*0.0*	*0.2*	*0.1*	*0.1*	*0.1*	*0.1*							*0.0*
201120_*C. neoformans* mutant	1.3	1.5	1.3	1.2	1.7	1.2	1.2	1.4	0.9	1.1	1.1	1.1	1.3	2.1	1.9
210122_*C. neoformans* mutant	1.3	1.6	1.3	1.3	1.7	1.3	1.2	1.4	0.6	1.2	1.1	1.1	1.2		1.8
210202_*C. neoformans* mutant	1.5	1.6	1.4	1.4	1.6	1.3	1.3	1.1	1.1	1.1	1.2	1.2	1.3		1.9
average C. neoformans mutant	*1.4*	*1.6*	*1.3*	*1.3*	*1.7*	*1.3*	*1.2*	*1.3*	*0.9*	*1.1*	*1.1*	*1.1*	*1.3*	*2.1*	*1.9*
st dev	*0.1*	*0.0*	*0.1*	*0.1*	*0.1*	*0.1*	*0.1*	*0.2*	*0.2*	*0.1*	*0.1*	*0.0*	*0.1*		*0.1*
201120_*C. neoformans* WT	1.6	2.0	1.2	1.5	1.8	1.5	1.5	1.6	1.7	1.3	1.5	1.5	1.9		2.1
201204_*C. neoformans* WT	1.6	2.2	1.7	1.4	1.6	1.4	1.4	1.7	1.6	1.3	1.6	1.1	2.5		1.9
210122_*C. neoformans* WT	1.7	1.8	1.1	1.6	1.5	1.5	1.6	1.7	1.3	1.3	1.2	1.4	1.7		1.9
210122_*C. neoformans* WT	1.7	1.7	1.0	1.4	1.5	1.4	1.4	1.2	1.4	1.3	1.3	1.3	1.7		2.0
210202_*C. neoformans* WT	1.8	1.8	1.3	1.6	1.6	1.7	1.4	1.8	1.4			1.4	2.1		
average C. neoformans WT	*1.7*	*1.9*	*1.3*	*1.5*	*1.6*	*1.5*	*1.5*	*1.6*	*1.5*	*1.3*	*1.4*	*1.3*	*2.0*		*2.0*
st dev	*0.1*	*0.2*	*0.3*	*0.1*	*0.1*	*0.1*	*0.1*	*0.2*	*0.2*	*0.0*	*0.2*	*0.2*	*0.4*		*0.1*

**Elemental atomic %**	**Na**	**O**	**N**	**C**	**K**	**Cl**	**S**	**P**
201120_*C. albicans*_yeast	0.2	40.2	3.0	56.2				0.4
201204_*C. albicans*_yeast	0.2	39.1	2.5	57.7	0.2			0.4
210202_*C. albicans*_yeast	0.4	37.4	2.7	59.0			0.06	0.5
average C albicans yeast	*0.3*	*38.9*	*2.7*	*57.6*	*0.2*			*0.4*
st dev	*0.1*	*1.4*	*0.3*	*1.4*				*0.0*
201113_2_*C. albicans* hyphae	0.1	42.8	3.0	53.7				0.4
210122_*C. albicans* hyphae	0.2	41.2	2.3	55.7			0.1	0.4
210202_*C. albicans* hyphae	0.3	40.1	1.7	57.5			0	0.4
average C albicans hyphae	*0.2*	*41.4*	*2.3*	*55.6*			*0.0*	*0.4*
st dev	*0.1*	*1.3*	*0.7*	*1.9*			*0.1*	*0.0*
201120_*C. neoformans* mutant	0.7	36.8	6.1	53.5	1.2	1.2		0.4

**% of total C**<br>**Spectral components**	**peptide**	**lipid**	**polysaccharide**
201120_*C. albicans*_yeast	23	10	67
201204_*C. albicans*_yeast	14	16	71
210202_*C. albicans*_yeast	20	12	69
average C albicans yeast	*19*	*13*	*69*
st dev	*5*	*3*	*2*
201113_2_*C. albicans* hyphae	20	13	67
210122_*C. albicans* hyphae	11	19	70
210202_*C. albicans* hyphae	29	0	71
average C albicans hyphae	*20*	*11*	*69*
st dev	*9*	*10*	*2*
201120_*C. neoformans* mutant	50	7	43

210122_*C. neoformans* mutant	1.3	52.0	5.3	37.2	1.4	2.4	0.4
210202_*C. neoformans* mutant	1.9	51.1	3.4	39.3	1.0	3.0	0.3
average C. neoformans mutant	*1.3*	*46.6*	*4.9*	*43.3*	*1.2*	*2.2*	*0.4*
st dev	*0.6*	*8.5*	*1.4*	*8.9*	*0.2*	*0.9*	*0.0*
201120_*C. neoformans* WT	0.8	41.3	3.6	53.3	0.5	0.3	0.2
201204_*C. neoformans* WT	1.1	48.2	5.8	43.7	0.3	0.6	0.3
210122_*C. neoformans* WT	1.1	61.8	2.5	33.5	0.3	0.6	0.1
210122_*C. neoformans* WT	0.7	56.5	2.9	39.1	0.3	0.3	0.1
210202_*C. neoformans* WT	1.4	42.0	2.7	53.2		0.4	
average C. neoformans WT	*1.0*	*50.0*	*3.5*	*44.6*	*0.4*	*0.4*	*0.2*
st dev	*0.3*	*9.0*	*1.3*	*8.7*	*0.1*	*0.2*	*0.1*

210122_*C. neoformans* mutant	59	10	31
210202_*C. neoformans* mutant	37	11	52
average C. neoformans mutant	*49*	*10*	*42*
st dev	*11*	*2*	*11*
201120_*C. neoformans* WT	51	0	49
201204_*C. neoformans* WT	61	0	39
210122_*C. neoformans* WT	50	0	50
210122_*C. neoformans* WT	50	0	50
210202_*C. neoformans* WT	42	0	58
average C. neoformans WT	*51*	*0*	*49*
st dev	*7*	*0*	*7*

**Ratio to total C**	**Na tot**	**O tot**	**N tot**	**C1**	**C2**	**C3**	**C4**	**K tot**	**Cl tot**	**S tot**	**P tot**
201120_*C. albicans*_yeast	0.004	0.715	0.054	0.205	0.612	0.183					0.007
201204_*C. albicans*_yeast	0.003	0.678	0.043	0.205	0.609	0.186		0.003			0.007
210202_*C. albicans*_yeast	0.006	0.635	0.045	0.204	0.581	0.215				0.001	0.008
average C albicans yeast	*0.004*	*0.676*	*0.047*	*0.205*	*0.601*	*0.195*		*0.003*			*0.007*
st dev	*0.002*	*0.040*	*0.006*	*0.001*	*0.017*	*0.018*					*0.001*
201113_2_*C. albicans* hyphae	0.003	0.797	0.056	0.203	0.593	0.204					0.007
210122_*C. albicans* hyphae	0.004	0.740	0.041	0.222	0.584	0.194				0.002	0.008
210202_*C. albicans* hyphae	0.004	0.697	0.029	0.152	0.622	0.227				0.000	0.007
average C albicans hyphae	*0.004*	*0.745*	*0.042*	*0.192*	*0.599*	*0.208*				*0.001*	*0.007*
st dev	*0.001*	*0.050*	*0.014*	*0.036*	*0.020*	*0.017*				*0.001*	*0.000*
201120_*C. neoformans* mutant	0.013	0.687	0.113	0.293	0.499	0.200	0.008	0.023	0.023		0.007
210122_*C. neoformans* mutant	0.034	1.395	0.144	0.380	0.408	0.205	0.007	0.038	0.064		0.011
210202_*C. neoformans* mutant	0.048	1.300	0.086	0.280	0.546	0.151	0.023	0.025	0.077		0.009
average C. neoformans mutant	*0.032*	*1.127*	*0.114*	*0.318*	*0.484*	*0.185*	*0.013*	*0.029*	*0.054*		*0.009*
st dev	*0.018*	*0.384*	*0.029*	*0.055*	*0.070*	*0.030*	*0.009*	*0.008*	*0.028*		*0.002*
201120_*C. neoformans* WT	0.015	0.774	0.068	0.204	0.473	0.248	0.074	0.010	0.006		0.005
201204_*C. neoformans* WT	0.025	1.103	0.132	0.248	0.439	0.282	0.031	0.008	0.014		0.007

210122_*C. neoformans* WT	0.033	1.844	0.073	0.203	0.536	0.215	0.047	0.009	0.018		0.004
210122_*C. neoformans* WT	0.018	1.445	0.074	0.224	0.540	0.164	0.072	0.009	0.008		0.003
210202_*C. neoformans* WT	0.026	0.790	0.050	0.180	0.491	0.272	0.057		0.007		
average C. neoformans WT	*0.023*	*1.191*	*0.080*	*0.212*	*0.496*	*0.236*	*0.056*	*0.009*	*0.011*		*0.005*
st dev	*0.007*	*0.456*	*0.031*	*0.026*	*0.043*	*0.048*	*0.018*	*0.001*	*0.005*		*0.002*

Table S3. Data comparing two fitting strategies of C1s for *C. neoformans* WT strain (restricted parameters highlighted in green). Both fitting approaches referenced the binding energy scale to 285.0 eV.

	alternative fitting					general fitting			
**Binding Energy (eV)**	**C 1s**	**C 1s**	**C 1s**	**C 1s**	**C 1s**	**C 1s**	**C 1s**	**C 1s**	**C 1s**
201120_*C. neoformans* WT	285.0	285.4	286.7	288.0	289.4	285.0	286.7	287.9	289.4
201204_*C. neoformans* WT	285.0	285.4	286.6	288.1	289.3	285.0	286.5	288.0	289.5
210122_*C. neoformans* WT	285.0	285.4	286.9	288.2	289.6	285.0	286.8	288.3	289.8
210122_*C. neoformans* WT	285.0	285.4	286.8	288.2	289.5	285.0	286.7	288.1	289.3
210202_*C. neoformans* WT	285.0	285.4	286.8	288.1	289.6	285.0	286.6	287.9	289.6
average C. neoformans WT	***285.0***	***285.4***	*286.8*	*288.1*	*289.5*	***285.0***	*286.6*	*288.0*	*289.5*
st dev			*0.1*	*0.1*	*0.1*		*0.1*	*0.1*	*0.2*
**FWHM (eV)**	FWHM restricted to maximum 1.4 eV					FWHM not restricted			
201120_*C. neoformans* WT	**1.4**	**1.4**	**1.4**	**1.4**	**1.4**	1.5	1.5	1.6	1.7
201204_*C. neoformans* WT	**1.4**	**1.3**	**1.4**	**1.4**	**1.4**	1.4	1.4	1.7	1.6
210122_*C. neoformans* WT	**1.4**	**1.4**	**1.4**	**1.4**	**1.4**	1.5	1.6	1.7	1.3
210122_*C. neoformans* WT	**1.4**	**1.4**	**1.4**	**1.3**	**1.4**	1.4	1.4	1.2	1.4
210202_*C. neoformans* WT	**1.4**	**1.4**	**1.4**	**1.4**	**1.4**	1.7	1.4	1.8	1.4
average C. neoformans WT	*1.4*	*1.4*	*1.4*	*1.4*	*1.4*	*1.5*	*1.5*	*1.6*	*1.5*
st dev	*0.0*	*0.0*	*0.0*	*0.1*	*0.0*	*0.1*	*0.1*	*0.2*	*0.2*
**Ratio to total C**									
201120_*C. neoformans* WT	0.145	0.072	0.467	0.234	0.082	0.204	0.473	0.248	0.074
201204_*C. neoformans* WT	0.196	0.062	0.451	0.235	0.056	0.248	0.439	0.282	0.031
210122_*C. neoformans* WT	0.111	0.110	0.492	0.211	0.076	0.203	0.536	0.215	0.047
210122_*C. neoformans* WT	0.134	0.091	0.535	0.170	0.069	0.224	0.540	0.164	0.072
210202_*C. neoformans* WT	0.091	0.072	0.535	0.218	0.084	0.180	0.491	0.272	0.057
average C. neoformans WT	*0.136*	*0.081*	*0.496*	*0.214*	*0.073*	*0.212*	*0.496*	*0.236*	*0.056*
st dev	*0.040*	*0.019*	*0.039*	*0.026*	*0.011*	*0.026*	*0.043*	*0.048*	*0.018*

Table S4. XPS data from phage replica including averages and standard deviations.

**Binding Energy Position (eV)**	**Na 1s**	**O1 1s**	**O2 1s**	**N1 1s**	**N2 1s**	**C1 1s**	**C2 1s**	**C3 1s**	**C4 1s**	**Cl 2p**	**Cl 2p**	**S 2p**	**S 2p**	**P 2p**
210119_Phi6 phage	1071.4	532.9	531.4	400.1	401.4	285.0	286.5	288.2	289.3	198.1	199.8			133.8
210119_2_Phi6 phage	1071.4	532.9	531.5	400.1	401.6	285.0	286.5	288.2	289.1	198.2	199.8	163.9	169.2	133.9
210126_Phi6 phage	1071.4	533.1	531.4	400.1	401.5	285.0	286.6	288.2	289.5	198.2	199.9			133.9
average Phi6	*1071.4*	*533.0*	*531.4*	*400.1*	*401.5*	*285.0*	*286.5*	*288.2*	*289.3*	*198.2*	*199.8*	*163.9*	*169.2*	*133.9*
st dev	*0.0*	*0.1*	*0.0*	*0.0*	*0.1*	*0.0*	*0.0*	*0.0*	*0.2*	*0.0*	*0.0*			*0.1*
210119_MS2 phage	1071.3	533.0	531.5	400.1	401.5	285.0	286.4	288.2	288.9	198.2	199.8	163.5	164.7	133.9
210119_2_MS2 phage	1071.3	533.1	531.5	400.0	401.5	285.0	286.4	288.2	289.2	198.2	199.8	164.0		133.8
210126_MS2 phage	1071.3	533.0	531.5	400.0	401.4	285.0	286.4	288.2	290.0	198.2	199.8	163.8		133.9
average MS2	*1071.3*	*533.0*	*531.5*	*400.0*	*401.5*	*285.0*	*286.4*	*288.2*	*289.4*	*198.2*	*199.8*	*163.8*		*133.9*
st dev	*0.0*	*0.0*	*0.0*	*0.0*	*0.1*	*0.0*	*0.0*	*0.0*	*0.6*	*0.0*	*0.0*	*0.2*		*0.1*

**FWHM**	**Na 1s**	**O1 1s**	**O2 1s**	**N1 1s**	**N2 1s**	**C1 1s**	**C2 1s**	**C3 1s**	**C4 1s**	**Cl 2p**	**Cl 2p**	**S 2p**	**S 2p**	**P 2p**
210119_Phi6 phage	1.3	1.6	1.4	1.3	1.7	1.2	1.2	1.2	1.1	1.3	1.1			1.8
210119_2_Phi6 phage	1.4	1.5	1.4	1.3	1.4	1.2	1.2	1.1	1.1	1.1	1.3	1.7	1.8	1.9
210126_Phi6 phage	1.3	1.6	1.3	1.3	1.5	1.2	1.2	1.2	0.7	1.1	1.2			1.9
average Phi6	*1.3*	*1.6*	*1.4*	*1.3*	*1.5*	*1.2*	*1.2*	*1.2*	*0.9*	*1.2*	*1.2*	*1.7*	*1.8*	*1.8*
st dev	*0.1*	*0.0*	*0.1*	*0.0*	*0.2*	*0.0*	*0.0*	*0.0*	*0.2*	*0.1*	*0.1*			*0.1*
210119_MS2 phage	1.4	1.9	1.4	1.4	**1.5***	1.2	1.4	1.1	1.2	1.3	1.2	1.2	1.2	2.0
210119_2_MS2 phage	1.4	1.8	1.4	1.4	**1.5***	1.3	1.3	1.1	0.9	1.3	1.3	2.0		1.9
210126_MS2 phage	1.4	2.0	1.4	1.4	1.8	1.3	1.4	1.4	0.4	1.3	1.4	1.8		1.9
average MS2	*1.4*	*1.9*	*1.4*	*1.4*	*1.6*	*1.3*	*1.4*	*1.2*	*0.9*	*1.3*	*1.3*	*1.7*		*1.9*
st dev	*0.0*	*0.1*	*0.0*	*0.0*	*0.2*	*0.0*	*0.0*	*0.1*	*0.4*	*0.0*	*0.1*	*0.4*		*0.1*

*restricted fitting FWHM max =1.5

Atomic % at the surface	Na	O	N	C	Cl	S	P	% of tot C<br>peptide	lipid	polysaccharide
210119_Phi6 phage	1.3	36.9	7.7	52.9	0.4	0.0	0.9	79	7	15
210119_2_Phi6 phage	1.2	37.0	7.8	52.6	0.4	0.2	0.8	82	5	13
210126_Phi6 phage	0.8	47.0	6.2	45.1	0.3		0.6	81	5	13
average Phi6	*1.1*	*40.3*	*7.2*	*50.2*	*0.4*	*0.1*	*0.8*	*48*	*24*	*28*
st dev	*0.3*	*5.8*	*0.9*	*4.4*	*0.1*	*0.1*	*0.1*	*5*	*5*	*0*
210119_MS2 phage	1.4	39.2	11.4	46.2	0.8	0.2	0.8	52	21	28
210119_2_MS2 phage	1.1	40.4	10.4	45.6	1.0	0.3	1.1	50	22	28
210126_MS2 phage	1.6	39.4	11.7	45.5	0.8	0.2	0.8	43	30	27
average MS2	*1.4*	*39.6*	*11.2*	*45.7*	*0.9*	*0.3*	*0.9*	*81*	*6*	*14*
st dev	*0.3*	*0.6*	*0.6*	*0.4*	*0.1*	*0.1*	*0.2*	*2*	*1*	*1*

Ratio to total C	Na /C	O /C	N /C	Cl /C	S /C	P /C	C1/Ctot	C2/Ctot	C3/Ctot	C4/Ctot
210119_Phi6 phage	0.024	0.697	0.145	0.007	0.000	0.016	0.408	0.396	0.178	0.017
210119_2_Phi6 phage	0.023	0.703	0.148	0.008	0.004	0.015	0.411	0.396	0.177	0.016
210126_Phi6 phage	0.017	1.042	0.136	0.007		0.014	0.443	0.370	0.176	0.011
average Phi6	*0.021*	*0.814*	*0.143*	*0.007*	*0.002*	*0.015*	*0.421*	*0.387*	*0.177*	*0.015*
st dev	*0.004*	*0.197*	*0.006*	*0.001*	*0.003*	*0.001*	*0.020*	*0.015*	*0.001*	*0.004*
210119_MS2 phage	0.031	0.849	0.248	0.017	0.005	0.018	0.420	0.347	0.181	0.052
210119_2_MS2 phage	0.024	0.885	0.229	0.023	0.007	0.024	0.434	0.324	0.223	0.019
210126_MS2 phage	0.036	0.867	0.256	0.018	0.004	0.017	0.432	0.248	0.317	0.003
average MS2	*0.030*	*0.867*	*0.244*	*0.019*	*0.006*	*0.020*	*0.428*	*0.306*	*0.241*	*0.025*
st dev	*0.006*	*0.018*	*0.014*	*0.003*	*0.001*	*0.004*	*0.007*	*0.052*	*0.070*	*0.025*
